# Adapting Atomic Configuration Steers Dynamic Half-Occupied State for Efficient CO_2_ Electroreduction to CO

**DOI:** 10.1021/jacs.5c03121

**Published:** 2025-04-01

**Authors:** Jiali Wang, Hui Ying Tan, Chia-Shuo Hsu, You-Chiuan Chu, Ching-Wei Chan, Kuan-Hsu Chen, Xuan-Rou Lin, Yi-Chun Lee, Hsiao-Chien Chen, Hao Ming Chen

**Affiliations:** † Department of Chemistry, National Taiwan University, Taipei 106, Taiwan; ‡ National Synchrotron Radiation Research Center, Hsinchu 300, Taiwan; § Center for Reliability Science and Technologies; Center for Sustainability and Energy Tecnhologies, Chang Gung University, Taoyuan 33302, Taiwan; ∥ Center for Emerging Materials and Advanced Devices, National Taiwan University, Taipei 10617, Taiwan

## Abstract

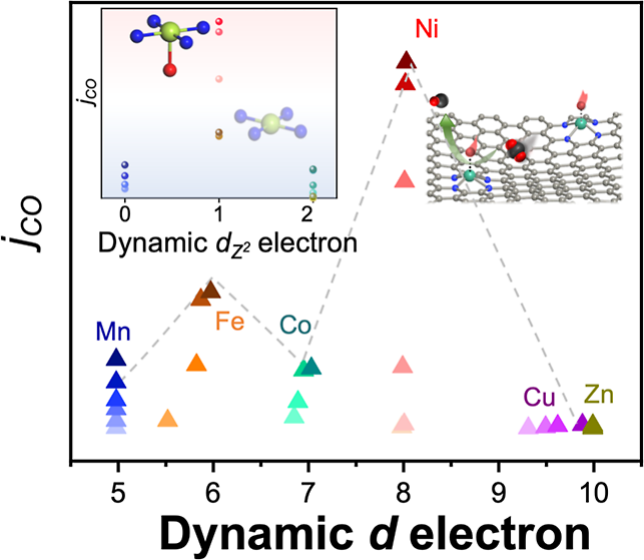

Electronic structures stand at the center to essentially understand the catalytic performance and reaction mechanism of atomically dispersed transition-metal–nitrogen–carbon catalysts (ADTCs). However, under realistic electrocatalytic conditions, the dynamic electronic disturbance at metal centers originating from complicated interactions with microenvironments is commonly neglected, which makes a true structure–property correlation highly ambiguous. Here, we employ operando time-resolved X-ray absorption spectroscopy to delve deeply into dynamic electronic behaviors of a family of transition-metal centers that are observed to adaptively vary in the metal–ligand configuration during the CO_2_ electroreduction reaction. We identify dynamic electronic/geometric configuration and d-orbital occupation under working conditions, demonstrating an unprecedentedly precise activity descriptor, i.e., dynamic axial *d*_*z*_^2^ electron, for the CO_2_-to-CO conversion. Direct results validate that the half-occupied state suggests the optimum binding behaviors with intermediates, significantly promoting CO production, which has been demonstrated by a significant kinetics enhancement of 1 to 2 orders of magnitude as compared with fully occupied and unoccupied states. This work presents the first empirical demonstration for a real correlation between the dynamic electronic/geometric configuration and catalytic kinetics in ADTCs, paving a new way for modulating catalysts and designing highly efficient reaction pathways.

## Introduction

Atomically dispersed transition-metal–nitrogen–carbon catalysts (ADTCs) benefit from the high atom efficiency and tunable structure analogous to molecular complexes, exhibiting great promise as cost-effective alternatives to commercial particle/bulk metal catalysts in heterogeneous electrocatalysis.^1−3^ For electrocatalytic carbon dioxide reduction reaction (CO_2_RR), a technology capable of converting CO_2_ into carbon-based chemicals and fuels using renewable electricity,^4,5^ ADTCs are particularly interesting due to their high activity and selectivity. It has been demonstrated that ADTCs are the most efficient in CO_2_-to-CO conversion depending on the favorable single metal sites with suppressed ability of C–C dimerization. To date, Mn, Fe, and Ni ADTCs have been reported to show a superior Faradaic efficiency and current density to state-of-the-art Au and Ag catalysts in laboratory cells.^1,2,6,7^ Moreover, an intriguing capability of Cu ADTCs to form C_2+_ products beyond CO was also reported.^8−10^ Despite the fact that many breakthroughs in performance have been made, it was observed that in numerous literature there are significant discrepancies in catalytic activity and/or selectivity on the similar single-metal moieties, which were synthesized via different approaches but sharing a key common structural feature, resulting in the intrinsic nature of improved catalytic performance remaining greatly debated. To address these disputes for guiding efficient ADTC design, establishing a universal theory, i.e., a realistic structure–property relationship, is highly imperative yet still not well disclosed.

Since those pioneering reports on ADTCs for the CO_2_RR, research has focused on identifying the atomic structure of active sites. Most works pointed out that the intrinsic catalytic efficiency of ADTCs for CO_2_RR has been intimately connected to the nature of active metal sites and their coordination structure features based on empirical and theoretical studies.^11,12^ Notably, observations of dynamic structural changes in catalytic sites that occur under working electrochemical conditions have raised critical concerns over the true nature of active centers,^13−15^ which calls for operando studies on ADTCs during CO_2_RR.^16,17^ A fairly representative investigation is provided by Cu ADTCs, where CO_2_ can be electrochemically converted into diverse products ranging from CO to multicarbon species on isolated Cu centers. Operando spectroscopy evidenced reduction of Cu^2+^/^+^ to metallic Cu^0^ sites in Cu ADTCs under cathodic CO_2_RR conditions, which further decreases their affinity for N-coordination and gives rise to Cu agglomeration; thus, the high selectivity toward multicarbon products was linked to the emergence of Cu_*n*_ clusters. However, for ADTCs incorporating metals other than Cu where the CO production dominates over the CO_2_RR, the degree of dynamic structural evolution is much lower than that of Cu ADTCs. It is noted that structural evolution of ADTCs under CO_2_RR conditions has only intensively been studied on Cu sites so far, while a systematic investigation of operando behaviors of other interesting metal centers beyond Cu has been substantially lacking. As a result, the precise active configuration for CO_2_-to-CO conversion on ADTCs remains poorly understood. Recently, a family of singly dispersed M–N–C (M = Mn, Fe, Co, Ni, Cu) catalysts were investigated in CO_2_RR and a volcano trend between the activity toward CO formation and the number of *d*-antibonding electrons was identified based on the reduced oxidation state of metal sites.^18^ Nonetheless, it is noted that the realistic changes in the local configuration of metal single sites under working conditions were overlooked when evaluating catalytic performance.^3,18^ In a further operando CO_2_RR study on Fe, Sn, Cu, Co, Ni, and Zn–N–C catalysts containing both metal single sites and varying degrees of metal oxide clusters,^19^ it demonstrated that the reversible cluster formation was ubiquitous in all catalysts, except for Ni–N–C, during electrocatalysis at −1.15 V_RHE_, highlighting different restructuring behaviors of metal sites in these materials as compared with single-atom electrocatalysts.

Unlike metal crystals, where the d-bandwidth/center of their continuous bands significantly affects the catalytic performance (d-band theory), the isolated local moieties in ADTCs create discrete orbitals of metal centers; thus, the energy level and symmetry of these orbitals that are strongly correlated with the atomic configuration of active metal sites essentially dictate the catalytic fate. Specifically, in ADTCs, metal ions have been stabilized by C, N, and O atoms in a square-planar geometry (*D*_4*h*_),^20^ where the 5-fold degenerate d bands split into a doubly degenerate *d*_π_ band (*d*_*xz*_ and *d*_*yz*_) as well as singly degenerate *d*_*xy*_, *d*_*z*^2^_, and *d*_*x*^2^_-_*y*^2^_ bands. Based on this orbital organization, catalytic performance can be understood by the Sabatier principle in terms of binding energies and energetic reaction paths, in which a favorable binding strength between metal sites and key reaction intermediates would endow a superior activity at the top of the volcano curve. Note that a comprehensive mechanistic understanding cannot solely depend on the resting d-orbitals of metal atoms, as isolated metal centers undergo continuous dynamic restructuring during the reaction.^13^ Under such situations, the hybridization between the d-orbital of the transition metals and the p-orbital of adjacent coordinated atoms can induce d-orbital reordering and electron redistribution within the frontier orbitals, dynamically influencing the binding energy of reaction intermediates.^16^ These insights emphasize the importance of in-depth operando studies to investigate the dynamic atomic configuration changes of active metal sites under the CO_2_RR conditions.

**Figure 1:**
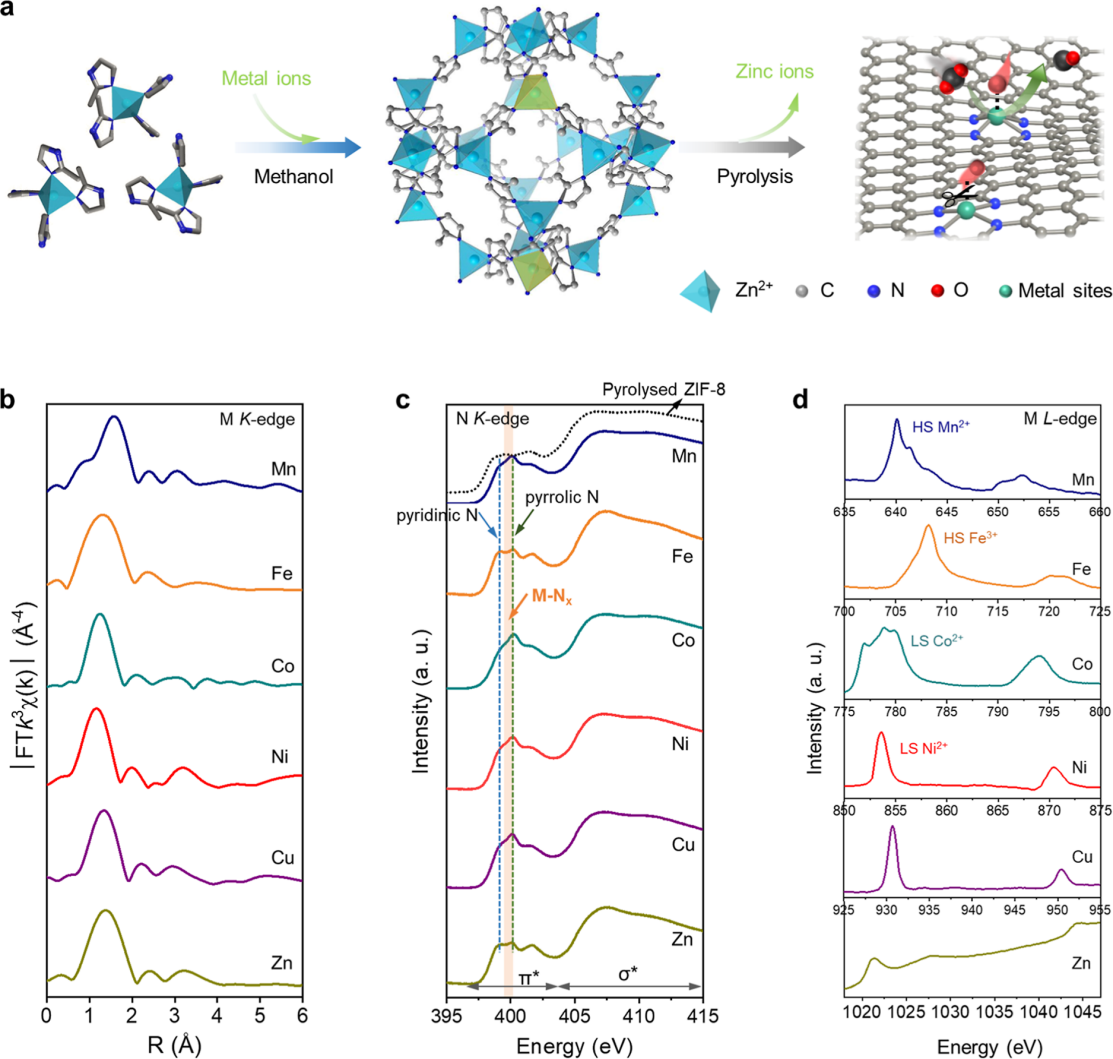
Structural characterizations of various ADTCs. (a) Schematic illustration of the formation of various ADTCs. (b) Fourier transformed (FT) *k*^3^-weighted metal K-edge extended X-ray absorption fine structure (EXAFS) spectra of various ADTCs. (c) N K-edge X-ray absorption near-edge structure (XANES) spectra of various ADTCs. (d) Metal L-edge XANES spectra of various ADTCs.

Motivated by the aforementioned results, we carry out here systematic operando studies on a series of ADTCs during the CO_2_RR to gain insights into a dynamically geometric and electronic configuration responsible for catalytic properties under working conditions. To this end, an entire family of zeolitic imidazolate framework (ZIF)-based metal single atoms (Mn, Fe, Co, Ni, Cu, and Zn) were prepared as model catalysts and employed operando time-resolved X-ray absorption spectroscopy (TR-XAS) to disclose realistic electronic/geometric configuration evolutions over the course of the CO_2_RR (Figure 1a). Significantly, electron transfer and distribution were comprehensively understood from a dynamic perspective based on operando spectra, rather than relying solely on static characterizations or theoretical DFT calculations. The findings reveal that a dynamic configuration-induced d-orbital reorganization and electron redistribution actively modulate metal centers, intrinsically regulating the electrocatalytic process. This study confirms that dynamic d-electrons along the axial direction serve as a precise activity descriptor for CO_2_RR kinetics, with a single electron occupation in the *d*_*z*_^2^ orbital, optimizing binding behavior and significantly enhancing CO production. Beyond establishing a robust structure–activity relationship for catalyst design, this work also clearly unfolds reasonable calculation models for future theoretical understandings, which contributes to realizing highly an efficient catalysis system.

## Results and Discussion

### Structural Characterizations of ADTCs

Synthesis of a family of ADTCs with well-defined metal sites is schematically illustrated in Figure 1a. A zeolitic imidazolate framework-8 (ZIF-8) was employed as an ideal framework providing C and N sources to anchor metal active centers. Various metal sites are introduced during the growth of ZIF nanocrystals by simultaneously adding transition-metal M ions (M = Mn, Fe, Co, Ni, and Cu). After a subsequent pyrolysis step, ADTCs with ML_*x*_ (L, coordinated atoms) moieties can be successfully anchored on the porous nitrogen-doped carbon substrate (see details in the Experimental Section). Zn in ZIFs is easily evaporated at a temperature above 900 °C, as supported by <0.1 wt % of the residual Zn content in the Mn, Fe, Co, Ni, and Cu ADTCs based on inductively coupled plasma-optical emission spectrometry (ICP-OES). By utilizing a relatively low-temperature pyrolysis procedure, Zn ADTC control sample was prepared by the same approach without addition of other transition-metal ions. The porous rhombic dodecahedron morphology is confirmed by transmission electron microscopy (TEM) and high-angle annular dark-field scanning TEM (HAADF-STEM) images (Figure S1). Brunauer–Emmett–Teller (BET) analysis reveals that the specific surface area of various ADTCs ranges from 386 to 539 m^2^ g^–1^ (Table S1), facilitating the exposure of more active sites for heterogeneous catalysis. TEM images further confirm the absence of any crystalline metallic phases in the catalysts, as validated by powder X-ray diffraction (XRD), in which the XRD patterns exhibit only two dominant peaks at 25° and 44°, corresponding to the characteristic diffractions of graphitic carbon (Figure S2). Raman spectra display broad D and G peaks, indicating graphitized carbon structures with similar defect levels across different ADTCs (Figure S3). Additionally, aberration-corrected HAADF-STEM imaging provides direct visualization of isolated metal sites in ADTCs (Figure S4). Energy-dispersive X-ray spectroscopy mapping reveals the homogeneous dispersion of C, N, and metal species throughout the entire ADTC architecture (Figure S1). The actual metal loading, determined via ICP-OES, ranges from 2.03 to 2.55 wt %, aligning with X-ray photoelectron spectroscopy (XPS) results (Table S2). This consistent metal content across ADTCs ensures a fair comparison of the catalytic activity in heterogeneous reactions.

To unambiguously reveal the single-atom nature and coordination structure of metal centers in ADTCs, the metal K-edge EXAFS is employed for in-depth investigations. The Fourier-transformed (FT) *k*^3^-weighted EXAFS spectra of the six ADTCs exhibit a dominant peak in the range of 1.2–1.56 Å (Figures 1b and S5), corresponding to the first coordination shell of light backscattering atoms, such as metal atoms coordinated to N or O. No characteristic metallic M–M peaks were observed in all samples, further confirming that the metal sites are atomically dispersed on the substrates. Quantitative structural information was obtained through first-shell fitting of the EXAFS spectra (Figure S6 and Table S3). The best-fit results indicate that Mn, Co, Ni, Cu, and Zn in ADTCs are primarily coordinated by four N atoms on nitrogen-doped carbon planes, suggesting that these metal cations are most likely involved in in-plane MN_4_ moieties. A notable exception is Fe-ADTC, which exhibits a significantly broadened first-shell peak. Further analysis revealed that in addition to the in-plane MN_4_ configuration, the presence of two axial light atoms was essential to achieve the best fit for the EXAFS spectrum. This finding suggests the formation of Fe–O axial pairs, which aligns with the high oxophilicity of Fe compared to other metals.^18,21^ Based on these results, it can be concluded that the as-prepared ADTCs contain only N, O-coordinated metal single-site moieties embedded in carbon materials, free from metal or oxide clusters.^19^

The electronic and atomic interplay of metal centers with coordinated atoms in ADTCs were investigated by XPS and synchrotron-radiation-based XANES spectroscopy. Deconvolution of N 1s XPS spectra for all ADTCs display four typical peaks that are assigned to pyridinic (398.6 eV), pyrrolic (400.3 eV), graphitic (401.3 eV), and oxidized N (403.6 eV) species (Figure S7). An additional peak situated at 399.4 eV implies coordination of N atoms with metal centers (M–N_*x*_), supporting the presence of M–N bonds in various ADTCs. In N *K*-edge XANES spectra, as compared with pyrolyzed ZIF-8, a remarkable intensity increase was observed for ADTCs in the energy range of 399.0–400.5 eV corresponding to pyrrolic N (Figure 1c), evidencing that metals sites are coordinated with pyrrolic-type N. The chemical states of central metal sites were first determined by the metal 2p_3/2_ XPS spectra (Figure S8) and *K*-edge XANES spectra (Figure S9). By referring to the binding and edge energies of various reference samples, it identifies +3 chemical state for the Fe site and +2 chemical state for Mn, Co, Ni, and Zn sites, while coexistence of Cu^2+^ and Cu^+^ species for Cu sites. Note particularly that the *K*-edge XANES spectra can be significantly varied depending on the coordination environment of metal sites;^22^ thus, a highly complementary characterization method is in high demand. Toward this end, metal *L*-edge XANES spectroscopy was utilized for achieving an accurate evaluation on both chemical and spin states of metal sites because it directly probes electronic transitions from 2p to unoccupied 3d valence band. As shown in Figure 1d, Mn L_2,3_-edge XANES spectrum of Mn ADTC exhibits similar characteristic peaks to the Mn^2+^ high-spin multiplet structure observed for MnO,^23^ indicating a high-spin Mn^2+^ electronic configuration in Mn ADTC, which has demonstrated a distorted planar geometry of Mn sites with four coordination atoms (*D*_4*h*_ symmetry) in the literature.^24^ Furthermore, Fe^3+^ sites in Fe-ADTC can be identified as existing in a high-spin state under a six-coordinated *D*_4*h*_ symmetry.^25^ In contrast, Co^2+^ in Co-ADTC adopts a low-spin state,^26^ Ni^2+^ in Ni-ADTC also exhibits a low-spin state,^27^ while Cu-ADTC contains a mixture of Cu^2+^ and Cu^+^ species,^28^ and Zn^2+^ is present in Zn-ADTC.^29^

### Dynamic Structural Configuration and Corresponding CO_2_RR Kinetics

**Figure 2:**
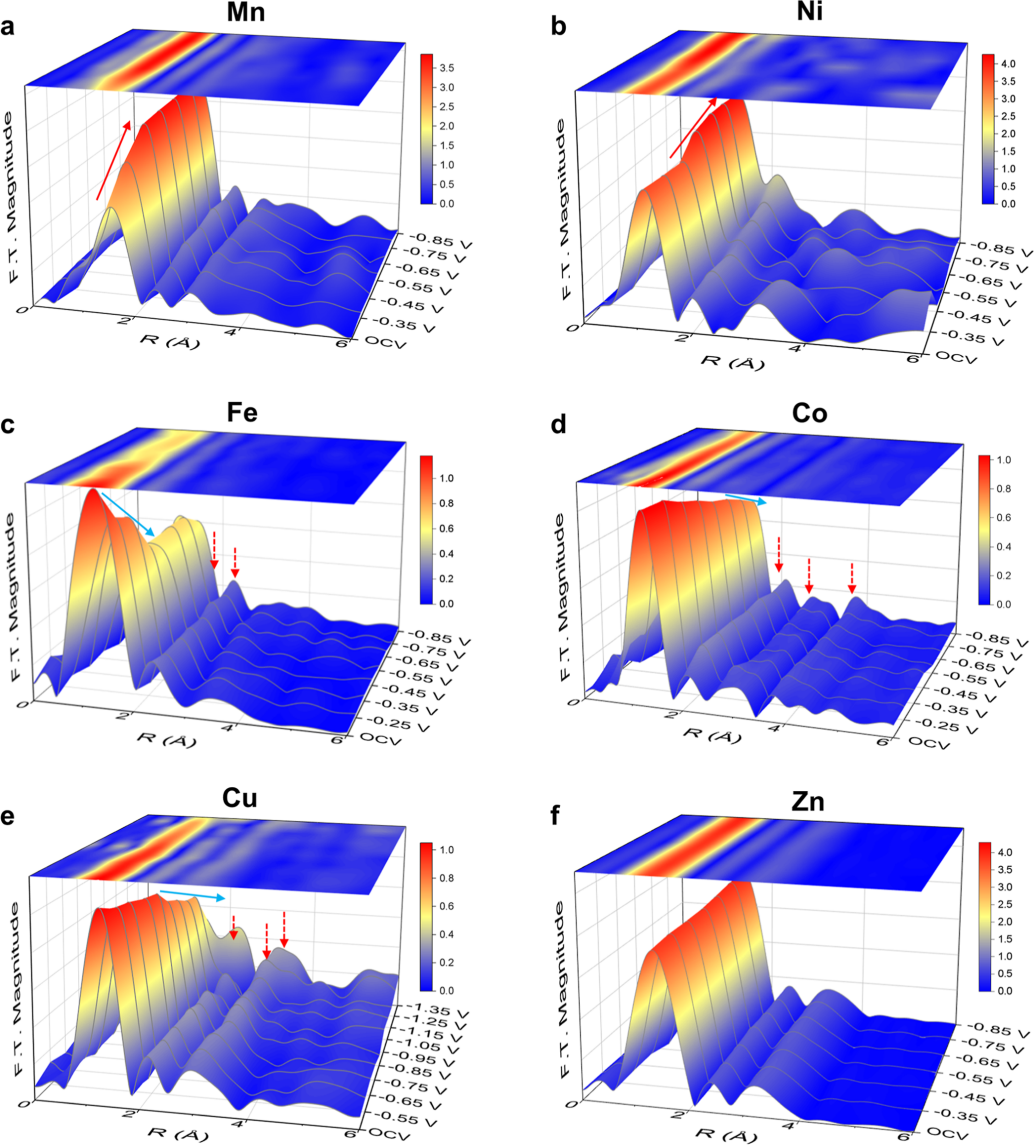
Dynamic structural evolutions during CO_2_RR. Operando time-resolved metal K-edge EXAFS spectra for (a) Mn, (b) Ni, (c) Fe, (d) Co, (e) Cu, and (f) Zn ADTCs under CO_2_RR conditions.

To realize a real-time probe of local structure evolutions of metal sites during CO_2_RR, we resorted to an advanced operando time-resolved XAS (TR-XAS) technique. As compared to the conventional XAS method that suffers from the poor temporal resolution due to a slow energy scan for incident X-ray source, the time-resolved XAS technique using a quick-scan monochromator enables rapid and continuous energy scans, which can realize real-time monitoring of rapid restructuring behaviors in catalytic reactions.^16,30,31^ TR-XAS spectra were collected with an acquisition rate of ∼50 s per spectrum, and operando metal *K*-edge FT-EXAFS spectra are shown in Figure 2. It is intriguing to observe the distinct evolutions of spectral features for different ADTCs with increasing cathodic potentials, suggesting that the local structure significantly varies with different ML_*x*_ sites over the CO_2_RR process. To be specific, for Mn, Ni, and Zn ADTCs, EXAFS spectra show a major peak at the first shell over the whole CO_2_RR course, with no peak belonging to the M–M bond, evidencing that those metal centers could maintain their single-site nature under working conditions. Specifically, for Mn- and Ni-ADTCs, the intensity of the main peak in the EXAFS spectra significantly increases with increasing applied cathodic potentials, which can be attributed to the formation of additional M–C/O bonds overlapping the original M–N bonds. The possibility of this feature arising from M–C bonds can be ruled out, as the presence of M–C bonds in the first coordination shell has been validated to be extremely low.^32^ From a thermodynamic perspective, this observation appears counterintuitive, as oxygen adsorbates on metal sites are generally unstable under highly cathodic potentials, according to the Pourbaix diagram.^18^ Notably, one should consider that several possible factors might contribute to the emergence of M–O bonds on the metal surface during CO_2_ reduction. First, oxygen-containing species present in the electrolyte may adsorb onto metal centers.^33,34^ Second, the increase in local pH near the catalyst surface, triggered by CO_2_ or H_2_O reduction, can influence the interaction between adsorbed hydroxide ions and reactive metal ions.^35−37^ Lastly, the kinetics of CO_2_RR or the competing hydrogen evolution reaction may be significantly faster than the reduction of oxidized metal species under working conditions,^19^ leading to the persistence of M–O bond features in Mn and Ni ADTCs within the studied potential range. Notably, oxygen binding to Mn sites occurs at much earlier potentials compared to Ni sites, which can be attributed to the significantly more negative standard reduction potential of Mn (−1.185 V vs SHE, SHE: standard hydrogen electrode) relative to Ni (−0.257 V vs SHE). Interestingly, no discernible changes in the FT-EXAFS spectra were observed for Zn ADTC, indicating that Zn isolated sites remain stable under CO_2_RR conditions, with no formation or cleavage of Zn–N/O bonds. In contrast, Fe, Co, and Cu ADTCs exhibit a gradual decrease in the intensity of the main FT-EXAFS peak at varying potentials, suggesting the occurrence of M–L bond breaking during CO_2_RR. This decreasing trend is particularly pronounced for Fe sites at earlier potentials, which is closely linked to the dynamic cleavage of initial Fe–O bonds, a characteristic behavior specific to Fe ADTC. More remarkably, the M–M scattering paths can be observed in FT-EXAFS spectra of Fe, Co, and Cu ADTCs under larger cathodic potentials, demonstrating that metallic clusters can extensively form under large cathodic potentials, which might significantly impact the product profile during CO_2_RR. For instance, the appearance of Cu–Cu scattering path has been previously reported as highly active sites for generating C_2+_ products during CO_2_RR.^8,38^

**Figure 3:**
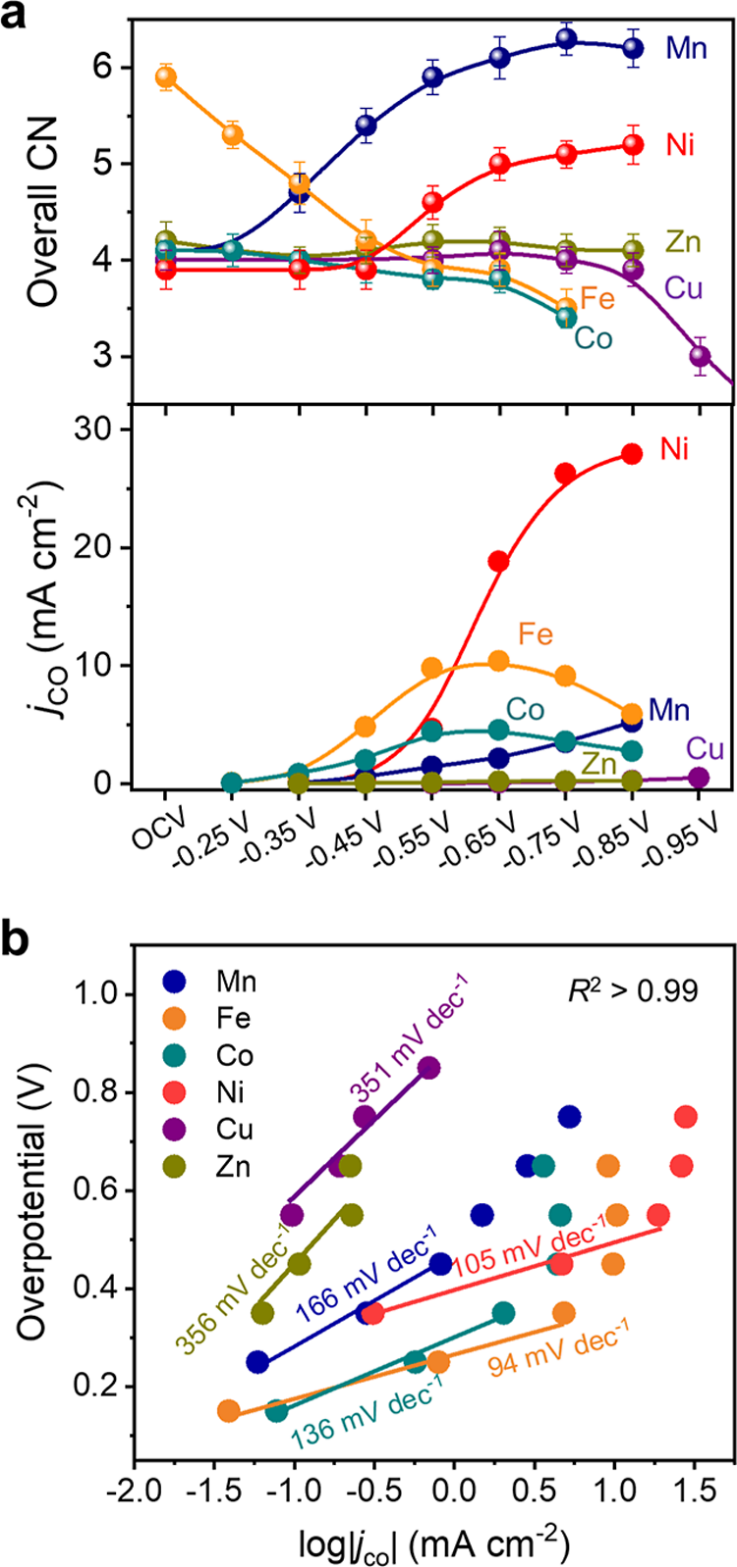
Electrocatalytic properties of CO_2_-to-CO conversion. (a) Correlation between the overall CN around various metal centers in ADTCs and partial current density of CO production at different applied potentials. (b) Tafel plots of various ADTCs toward CO production during CO_2_RR.

The discrepancy in dynamic configuration evolution of various ADTCs can be quantitatively evaluated based on the best fitting results of EXAFS spectra (Figures S10–S16 and Tables S4–S9). As shown in Figure 3a, upon application of cathodic potentials, the overall coordination number (CN) around Mn sites at the first shell immediately increases with a gradual trend from initial four- to six-coordination at −0.55 V_RHE_, while that of Ni sites shows an obvious increase at more cathodic potentials and reaches the maximum value of ∼ 5 at −0.65 V_RHE_, suggesting that under CO_2_RR conditions the atomic structure is most likely to transform from an initial square-planar configuration into a distorted octahedral and a square-pyramidal coordination in Mn and Ni ADTCs, respectively. Significantly, it was observed that the enhancement of CO partial current density, as referred to the kinetics of CO formation, exhibits a strong correlation with the increase in CN around Mn/Ni sites, indicating that the M–N_4_ moieties with additional M–O bond formation in Mn and Ni ADTCs are actually active sites toward CO_2_-to-CO conversion. This phenomenon is consistent with a recent observation of an oxygen-bridge adsorption onto the Ni_2_–N_6_ site that was validated to significantly lower the energy barrier for CO_2_-to-CO conversion.^39^ In the cases of Fe, Co, and Cu ADTCs, one can find that as the applied potential was extended to larger cathodic values (−0.75 V_RHE_ for Fe and Co and −0.95 V_RHE_ for Cu), the overall CN values of all metal sites show a decreasing trend, which is below the four coordination. Notably, for the case of Fe ADTC, it was also observed that a significant decline of the first-shell coordination upon the cathodic potentials were applied, while for Co and Cu ADTCs, the first coordination remains unchanged at potentials below −0.65 and −0.85 V_RHE_, respectively. The subsequent decrease of CN values validates the dynamic breaking behaviors of metal sites with neighboring ligands during CO_2_RR, in which only Fe–O bond cleavage occurs at small cathodic potential, while at considerably large cathodic potentials, M-N bonds in all ADTCs start to break and metallic clusters are most likely to form. By integrating kinetics of CO formation with dynamic configuration of metal sites, one can be inferred that as compared to Mn and Ni ADTCs, the four-coordinated Fe and Co, and lowered coordinated Cu sites are responsible for the enhanced kinetics of CO production. It is worth noting that Fe and Co metallic clusters have been evidenced as highly active sites for hydrogen evolution that is competitive to CO_2_RR,^40,41^ which can well explicate the depressed trend in catalytic kinetics of CO production at more cathodic potentials (<−0.65 V_RHE_). In addition, unlike the dynamic restructuring behaviors in the aforementioned two ways, the overall CN around Zn sites in Zn ADTC remains four in the whole potential window with a negligible kinetic enhancement, evidencing that the pristine Zn–N_4_ configuration is inefficient in converting CO_2_ for CO.

Based on the above results, a picture can be evidently depicted for the dynamic structure–activity correlation for a series of Mn, Fe, Co, Ni, Cu, and Zn ADTCs, which unequivocally unravels three categories of local structural evolutions of metal sites in dictating the catalytic kinetics of CO production (Figures 3a and S17–S20). First, Mn and Ni ADTCs undergo atomic structural transformation, forming a saturated coordination structure around the metal sites through axial oxygen bonding. This process facilitates a monotonic increase in the catalytic kinetics across the investigated potential range. Second, Fe and Co ADTCs initially maintain a four-coordinate configuration at small cathodic potentials but subsequently undergo dynamic bond cleavage, leading to the formation of metallic clusters at larger cathodic potentials. This structural evolution results in a volcano-shaped trend in the CO production rate as a function of the applied potential. Finally, Cu and Zn ADTCs retain their initial configurations throughout the investigated CO_2_RR potential range, exhibiting very limited catalytic activity for CO_2_-to-CO conversion. The distinct CO_2_-to-CO conversion kinetics observed for different ADTCs are further corroborated by Tafel analysis (Figure 3b). It reveals that Fe and Co ADTCs show the relatively low Tafel slopes at small overpotentials (<350 mV), while Mn and Ni ADTCs exhibit lower Tafel slopes at relatively large overpotentials (350–550 mV), which are significantly lower than those of Cu and Zn ADTCs, indicating their faster reaction kinetics at different potential ranges. It is particularly noted that the CO formation rate of Ni sites is five times higher than that of the prominent Fe ADTCs and even shows one-2 orders of magnitude as compared with that of Co, Cu, and Zn sites at −0.85 V_RHE_, exhibiting its outstanding capability in achieving industrially relevant current densities. As the present series of ADTCs has, except for the distinctive metal center with varied metal–ligand coordination structures, a negligible difference in morphology and composition, the distinct reaction kinetics for CO_2_-to-CO conversion can be ascribed to the dynamic three-dimensional metal–ligand interactions in the specific configuration under CO_2_RR conditions.

### Dynamic Electronic Structures behind Metal–Ligand Interactions during CO_2_RR

**Figure 4:**
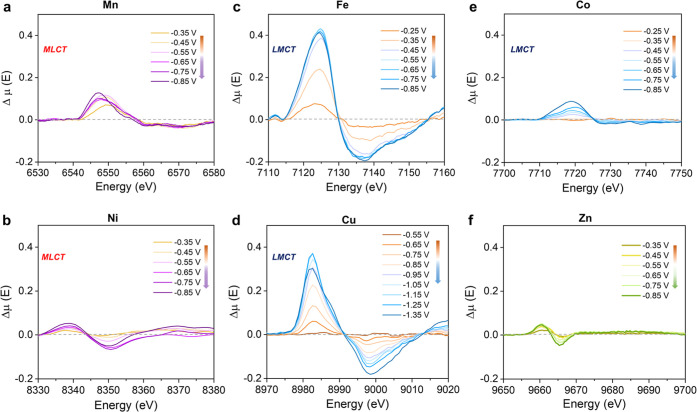
Dynamic electronic behaviors of various metal sites in ADTCs during CO_2_RR. Δμ analysis of operando time-resolved metal K-edge XANES spectra for (a) Mn, (b) Ni, (c) Fe, (d) Cu, (e) Co, and (f) Zn ADTCs under CO_2_RR conditions. Δμ analysis presents a subtraction methodology according to Δμ = μ(applied potential) – μ(OCV). MLCT: metal-to-ligand charge transfer, LMCT: ligand-to-metal charge transfer.

The above results stimulate us to investigate the underlying electronic behaviors behind the dynamic metal–ligand interactions for various ADTCs, which actually determine the catalytic fate toward CO_2_RR. Accordingly, operando *K*-edge XANES spectroscopy is a powerful tool, in which the spectra are characterized by intense white line features that result from splitting of 4p orbitals of metals due to hybridization between central metal atoms and neighboring ligands, thus providing insightful information about dynamic charge transfer and metal–ligand interactions.^42−47^ In contrast to the intuitive understanding that under cathodic potentials the absorption edge usually shifts toward lower energies and the white line is strongly suppressed, one can observe distinctly different evolutions of these spectral features for various ADTCs during CO_2_RR (Figures 4, S21 and S22), suggesting a special electronic configuration and transfer behaviors of metal sites in response to a dynamic environment. Generally, the white line of XANES spectra is split into two bands, which corresponds to the electron transition from the 1s to 4p_*z*_ orbital at relatively lower energies and from the 1s to 4p_*x*,*y*_ orbitals at high energies. For the case of Mn ADTC, as the applied potential was extended to larger cathodic potentials, the peak at 6553 eV, assigned to 1s → 4p_x, y_ transition, was observed to show an intriguing increase in intensity, demonstrating the elevation of 4p_*x*, *y*_ orbitals of Mn during CO_2_RR. From further Δμ analysis of XANES spectra (a subtraction methodology according to Δμ = μ(applied potential) – μ(OCV)), a remarkable positive-peak feature strengthens and shifts toward lower energies (Figure 4a), which indicates a less extent of splitting of the Mn 4p orbitals. These observations demonstrate the nature of a promoted electron transfer from metal to ligand orbitals, namely, metal-to-ligand charge transfer (MLCT), for Mn sites during the CO_2_RR, which induces the formation of additional axial coordination to the Mn–N_4_ planar configuration. Meanwhile, no absorption probability difference at the pre-edge energy (1s → 3d transition) was observed, further validating that the Mn sites with a centrosymmetric six-coordinated symmetry can well retain +2 chemical states under CO_2_RR conditions, which is in agreement with results of the first derivative of operando XANES spectra (Figure S22). Notably, a dynamic spin-transition in Mn sites was validated by the post-test Mn L-edge spectrum (Figure S23), where the decreased branching ratio of *L*_3_-edge intensity to total line strength (*I*(L_3_)/*I*(L_3_ + L_2_)) was observed after CO_2_RR,^48^ suggesting that Mn^2+^ sites dynamically evolve from high spin to low spin during the reaction. For Ni ADTC, operando XANES spectra show the narrowing and weakening of the white line corresponding to the Ni 4p_*x*, *y*_ orbitals, while the strengthening and positive shift of the positive-peak feature at the 1s → 4p_*z*_ region (Figure 4b), which demonstrates that the splitting of Ni 4p orbitals is reduced with the elevated 4p_*z*_ orbital, resulting in the MLCT character similar to Mn. A close inspection further finds the enhanced intensity on the pre-edge peak (originating from the 1s → 3d transition) and a negligible edge shift (Figure S22), evidencing formation of square-pyramidal Ni^2+^ sites, because a noncentrosymmetric distortion allows 3d–4p mixing that increases the intensity of the pre-edge feature.^49^ Such a square-pyramidal configuration of Ni^2+^ sites favors a high-spin state.^50^ Operando EXAFS spectra and fitting results reveal that the radial distance of Mn–L coordination gradually decreases, while that of Ni–L coordination increases with increasing cathodic potentials, which support a dynamically formed low-spin Mn^2+^ and high-spin Ni^2+^ configuration, respectively (Figure S10 and S13, Table S4 and S7).^51^ It is also worth noting that metal 4p_*x*,*y*_ orbitals are closely correlated with the states of metal–nitrogen bonding;^52^ thus, the highly situated 4p_*x*,*y*_ orbitals observed in both Mn and Ni ADTCs imply less electron population in antibonding orbitals, favoring their stable configuration during the reaction.

Notably, more profound XANES spectral changes were found for Fe and Cu ADTCs during CO_2_, it reveals the broadening and weakening of the white line (7120–7150 eV for Fe and 8980–9010 eV for Cu) during the cathodic potential excursion (Figure 4c,d), suggesting a downshift of 4p orbitals and a greater extent of splitting between 4p_*x*,*y*_ and 4p_*z*_ orbitals. This observation highlights a tendency of the ligand-to-metal charge transfer (LMCT) and increasing possibilities to destabilize the structural configuration. Moreover, one can find the increasingly negative shift of the pre-edge peak (1s → 3d transition) for Fe ADTC, which confirms the reduced nature of Fe sites as CO_2_RR proceeds. Further combining operando K-edge and post-test L-edge XANES spectra (Figures S22 and S24), we can conclude that the initial high-spin Fe^3+^ sites are dynamically evolving into high-spin Fe^2+^ states over the course of CO_2_RR. It is also noted that at potentials more anodic than −0.45 V_RHE_, significant spectral changes were observed for Fe ADTC, indicating the remarkable structure transformation as a result of cleavage of initial Fe–O bonds under electrochemical conditions, which is in agreement not only with previous reports but also with operando EXAFS results.^53,54^ Furthermore, the spectral features of Fe sites changed slightly within the potential range of −0.45 to −0.65 V_RHE_, similar to that of Co^2+^ sites in Co ADTC with a *D*_4*h*_ symmetry (Figure 4e), supporting the stable electronic configuration of Fe and Co sites toward the CO_2_RR within this potential range. Further increasing applied cathodic potentials, the continuous decrease of white line intensity accounts for the gradual breaking behavior of Fe/Co–N bonds and subsequent formation of metal clusters. Remarkably, for Cu ADTC, it was observed that the intensity of peak around 8983 eV (1s → 4p_*z*_ transition) significantly increases once the applied potential is more cathodic than −0.85 V_RHE_, which is consistent with spectral features of low-coordinated configuration.^30,55^ Such a peak attenuates at larger cathodic potentials, e.g., −1.35 V_RHE_, indicating the disappearance of low-coordinated configuration due to gradual formation of Cu clusters. These results demonstrate that the dynamic electronic interactions dictate metal–ligand configurations for Fe, Co, and Cu ADTCs during the CO_2_RR. Like neither those of Mn, Ni ADTCs nor Fe, Co, Cu ADTCs, operando XANES spectra of Zn ADTC exhibit quite weak changes during the CO_2_RR, where the chemical state was observed to be slightly reduced, demonstrating the insignificant electron fluctuations of Zn centers toward the CO_2_RR.

### New Insights into CO_2_RR Mechanism at Isolated Transition-Metal Sites

**Figure 5:**
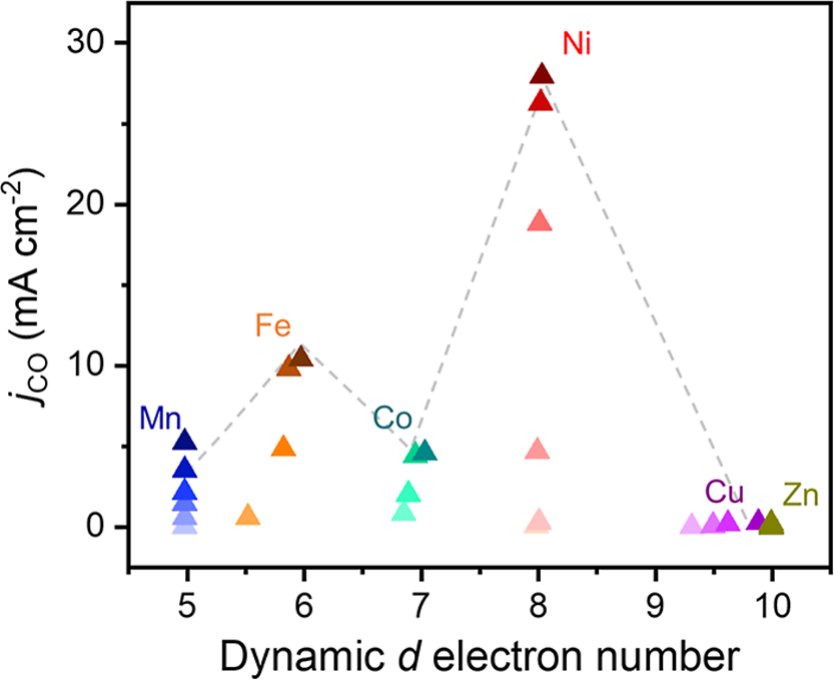
Correlation between dynamic d orbital and CO kinetics. A double volcano-shaped relationship between the electron numbers in dynamic d orbitals and CO partial current density. *j*_CO_ is evaluated with progressively cathodic potentials, color-coded from light to dark. The potential range is −0.35 to −0.85 V_RHE_ for Mn, Ni, and Zn, −0.35 to −0.65 V_RHE_ for Fe and Co, and −0.55 to −0.85 V_RHE_ for Cu. Potential interval: 0.1 V. The upper potential bounds chosen in this figure represent the highest potential that the atomic configuration of metal sites was able to sustain without rupture of M–N bonds and formation of metal clusters.

Given the dynamic electronic/geometric configuration of various metal sites during the reaction, we begin by examining the relationship between the dynamic d electron of metal centers and the catalytic kinetics of CO_2_-to-CO conversion for clarifying the authentic CO_2_RR mechanism at ADTCs. Based on the operando data discussed above, it is worth noting that increasing the applied cathodic potentials can obviously increase the d electron number for Fe and Cu atoms, while a relatively stable d electron number for Mn, Co, Ni, and Zn centers was observed (Figure 5). By combining with electrocatalytic results, it is evident to validate a double volcano-shaped relationship between the dynamic d electron number and CO partial current density, where Fe and Ni metal sites, respectively, having 6 and 8 electrons in d orbitals are situated at the peak position with the optimized kinetics for CO production. Nonetheless, it should be noted that the Ni site with a similar d electron number at various applied cathodic potentials exhibits quite different reaction kinetics for CO production, which further inspires us to identify a more accurate descriptor for CO_2_RR kinetics on ADTCs.

**Figure 6:**
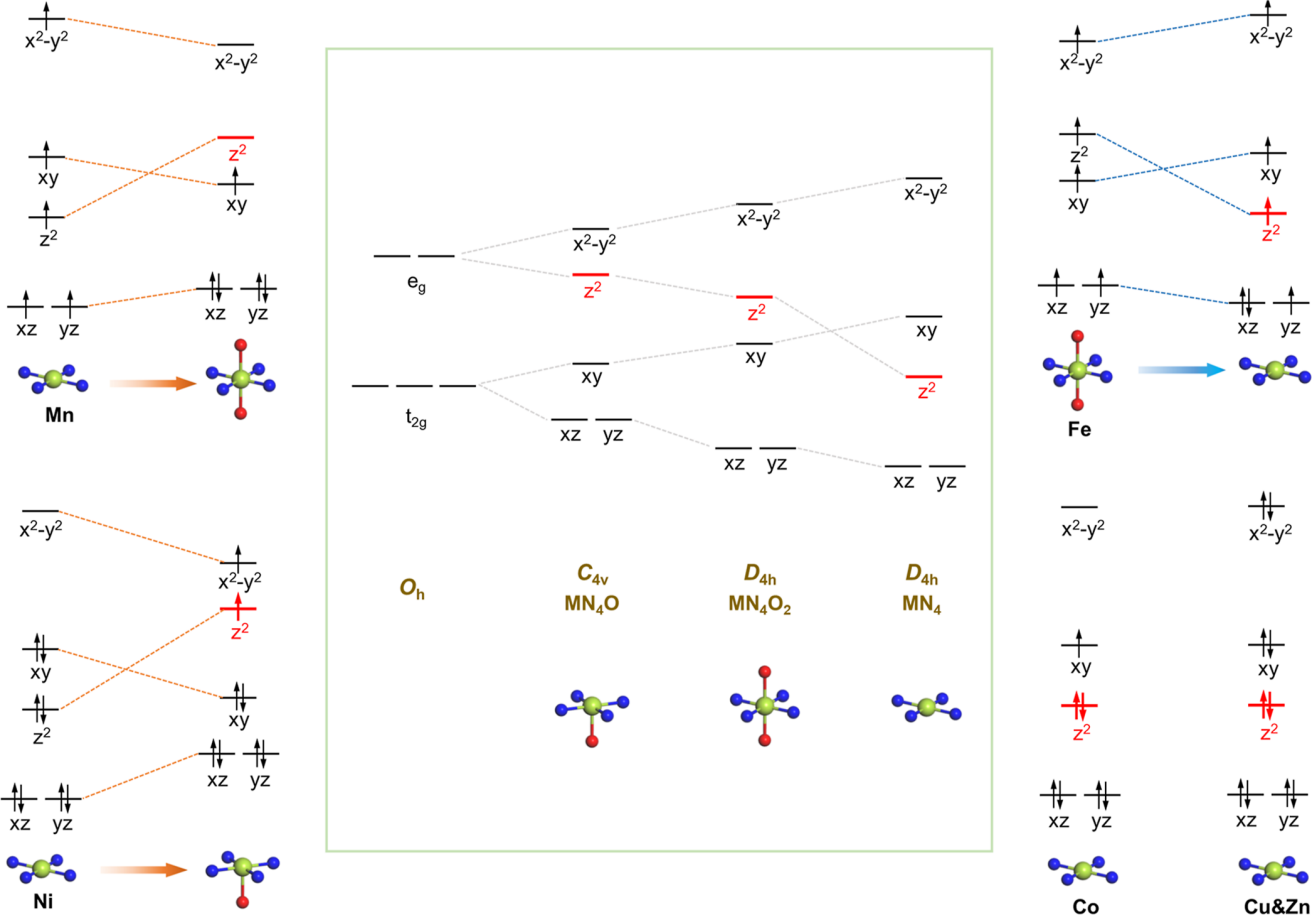
Dynamic orbital understandings of various transition-metal sites during CO_2_RR. Dynamic 3d orbital reorganization and electron filling in terms of various metal–ligand interaction configuration evolutions for ADTCs during CO_2_RR.

**Figure 7:**
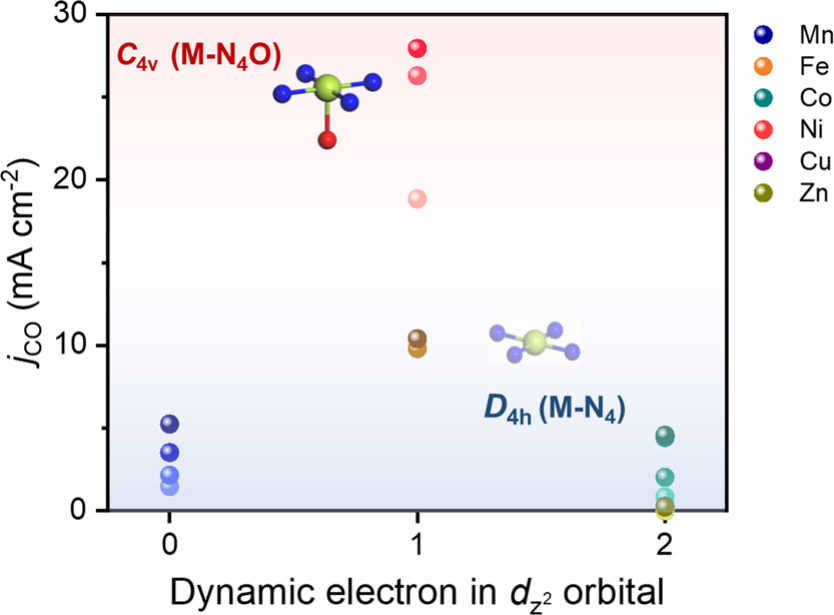
Proposed activity descriptor toward CO_2_RR. CO partial current density of various ADTCs as a function of the dynamic *d*_*z*_^2^ orbital occupation. The *j*_CO_ data with progressively cathodic potentials, color-coded from light to dark, are extracted from Figure 5. The potential range is −0.55 to −0.85 V_RHE_ for Mn, −0.55 to −0.65 V_RHE_ for Fe, −0.35 to −0.65 V_RHE_ for Co, −0.65 to −0.85 V_RHE_ for Ni, −0.55 to −0.85 V_RHE_ for Cu, and −0.35 to −0.85 V_RHE_ for Zn. Potential interval: 0.1 V. The lower potential bounds represent the lowest potential that the metal sites obtain the dynamically stable oxidation/spin states and atomic configuration during CO_2_RR, and the upper potential bounds represent the highest potential that the atomic configuration of metal sites was able to sustain without rupture of M–N bonds and formation of metal clusters.

In light of the crystal field theory, the dynamic local geometric symmetry greatly influences the molecular orbital degeneracy and sequence of isolated metal centers, which eventually manipulates their catalytic fate via a restructured electronic configuration. Based on all results discussed above, we can hypothesize that the metal 3d orbitals undergo dynamic restructuring as a result of the interaction between transition-metal sites and coordinated ligands/reactants with specific configurations during the reaction. Consequently, the dynamic energy level rearrangement and electronic filling of d orbitals that contribute to the frontier molecular orbitals of metal sites were further scrutinized during CO_2_RR. According to operando spectral evidence, the initial square-planar M–N_4_ sites of various ADTCs (except for Fe) with a *D*_4*h*_ symmetry exhibit distinctly different evolutions during CO_2_RR, i.e., from square-planar high-spin Mn^2+^ to low-spin Mn^2+^ sites with a distorted octahedral configuration (*D*_4*h*_ symmetry) for Mn ADTC and from square-planar low-spin Ni^2+^ to a high-spin Ni^2+^ site with a square-pyramidal configuration (*C*_4*v*_ symmetry) for Ni ADTC. Intriguingly, for Fe and Co ADTCs, within the potential range where the catalytic kinetics is monotonically increased (Figure 3a), we validate that the Fe site evolves from high-spin Fe^3+^ with a six-coordinated *D*_4*h*_ symmetry to high-spin Fe^2+^ in a four-coordinated *D*_4*h*_ symmetry, while the Co site retains its low-spin Co^2+^ states in a *D*_4*h*_ symmetry. Notably, Cu sites are prone to form reduced states in Cu ADTC before the structure is significantly changed from a single-atom configuration to metallic clusters. For Zn ADTC, except for slightly reduced states, Zn sites well retain the initial Zn–N_4_ configuration over the whole course of CO_2_RR. These operando findings allow a precise evaluation on the dynamic electronic filling within d orbitals of transition-metal sites and then the binding behaviors during CO_2_RR. As illustrated in Figure 6, a fact can be clearly revealed that the most profound feature comes from the electronic population in the *d*_*z*_^2^ orbital for various metals. It reveals an unoccupied *d*_*z*_^2^ orbital for Mn ADTC, one electron-occupied *d*_*z*_^2^ orbital (unpaired *d*_*z*_^2^ orbital) for Ni and Fe ADTCs, and two-electron-occupied *d*_*z*_^2^ orbital for Co, Cu, and Zn ADTCs under CO_2_RR working conditions. As is well known, the M-C (metal-reactant) bonds during CO_2_RR are dominantly contributed from interactions between the *d*_*z*_^2^ orbital of transition-metal sites and π* orbital of carbon-related molecules.^56^ Hence, the adsorption behaviors of metal sites toward the CO_2_RR can be well rationalized from the electronic occupation of the *d*_*z*_^2^ orbital. Significantly, plotting the CO_2_-to-CO kinetics as a function of the *d*_*z*_^2^ occupation of metal sites does produce a volcano relationship, as shown in Figure 7. It demonstrates that Fe and Ni having the moderate *d*_*z*_^2^ occupation of one electron exhibit remarkably enhanced kinetics during CO_2_RR, suggesting that the unpaired axial electron facilitates the optimum binding energy of metal sites with intermediates, which accounts for the outstanding capability of Fe and Ni ADTCs in promoting CO yield. The down trend of the volcano plot can be explained as follows. A lack of *d*_*z*_^2^ occupation (*d*_*z*_^2^ = 0) in the Mn–N_4_O_2_ configuration indicates strong binding with oxygen ligands, which hinders the adsorption of carbon-related intermediates for subsequent reactions. Conversely, a fully occupied *d*_*z*_^2^ orbital (*d*_*z*_^2^ = 2) in Co, Cu, and Zn sites results in excessively weak binding capabilities, also leading to limited CO_2_ conversion kinetics. Notably, these findings confirm that a square-pyramidal Ni^2+^ configuration represents an optimal system for achieving rapid CO formation during the CO_2_RR. This work provides the first empirical and explicit demonstration of dynamic *d*-state behavior under working conditions. It establishes an authentic CO_2_RR mechanism and identifies a precise activity descriptor—half-occupied *d*_*z*_^2^ electron—for isolated transition-metal centers, paving the way for a fundamental structure–activity relationship and the rational design of efficient catalysts.

### Conclusions

This work delves into the significant issue of dynamic d orbital behaviors of transition-metal sites as a result of adaptively varied metal–ligand interactions under working CO_2_RR conditions, which has been seriously neglected in the community but is key to accurately understand the reaction mechanism and efficiently design a catalytic system. Through resorting to operando time-resolved XAS analyses, direct spectral results evidenced distinctly different configuration evolutions of a family of isolated transition-metal sites during CO_2_RR, which causes dynamic reordering and electron redistribution in the frontier orbitals, allowing one to establish an imperative correlation between the dynamic d electron in axial with the reaction kinetics toward CO_2_-to-CO conversion. Significantly, we demonstrated that an electron filling in the *d*_*z*_^2^ orbital serves as a precise indicator of the fast reaction kinetics of CO production, which is linked to the optimum binding energies of metal centers. In this way, a square-pyramidal Ni^2+^ configuration was validated as the most promising candidate catalyst system for achieving industrially relevant current densities. This unprecedented understanding of the dynamic d orbital-dictated reaction kinetics in transition-metal-catalyzed CO_2_RR paves the way for the design and optimization of catalysts in electrochemical reactions.

## Supporting Information Available

Experimental details, materials, methods, STEM images, XPS data, in situ XANES spectra, and in situ EXAFS spectra, including the fitting results (PDF)
