# Supplementary material for: Adapting Atomic Configuration Steers Dynamic Half-Occupied State for Efficient CO2 Electroreduction to CO
Source: J Am Chem Soc. 2025 Apr 1;147(15):13027–38. doi: 10.1021/jacs.5c03121 (PMC12006994; doi:10.1021/jacs.5c03121)
Supplement: Supplementary file 1 — ja5c03121_si_001.pdf [file ja5c03121_si_001.pdf]

Supporting Information

Adapting Atomic Configuration Steers Dynamic Half-Occupied State for Efficient CO_2 Electroreduction to CO

Jiali Wang,[1†] Hui Ying Tan,[1†] Chia-Shuo Hsu,[2] You-Chiuan Chu,[1] Ching-Wei Chan,[1] Kuan-Hsu Chen,[1] Xuan-Rou Lin,[1] Yi-Chun Lee,[1] Hsiao-Chien Chen,[3] Hao Ming Chen[1,2,4]*

[1]Department of Chemistry, National Taiwan University, Taipei 106, Taiwan

[2]National Synchrotron Radiation Research Center, Hsinchu 300, Taiwan

[3]Center for Reliability Science and Technologies; Center for Sustainability and Energy Tecnhologies, Chang Gung University, Taoyuan 33302, Taiwan

[4]Center for Emerging Materials and Advanced Devices, National Taiwan University, Taipei 10617, Taiwan

[†]J. W. and H.-Y. T. contributed equally to this paper

Email: haomingchen@ntu.edu.tw

Experimental Section

Chemicals and materials: Manganese nitrate tetrahydrate ($Mn(NO_3)_2{\cdot}4H_2O$), ferric nitrate nonahydrate ($Fe(NO_3)_3{\cdot}9H_2O$), cobalt nitrate hexahydrate ($Co(NO_3)_2{\cdot}6H_2O$), nickel nitrate hexahydrate ($Ni(NO_3)_2{\cdot}6H_2O$), copper nitrate hydrate ($Cu(NO_3)_2{\cdot}3H_2O$), zinc nitrate hexahydrate ($Zn(NO_3)_2{\cdot}6H_2O$) and 2-methylimidazole ($C_4H_6N_2$) were purchased from Acros Organics. Nafion solution (20 wt%) and methanol (CH_3OH) were obtained from Sigma-Aldrich. The N_2 and CO_2 gases (99.999%) were purchased from Shen-Yi Gas Co. All chemicals were of analytic grade and used without further purification. Deionized (DI) water with the specific resistance of 18.25 MΩ·cm was obtained from an ELGA ultrapure water system.

Synthesis of various ADTCs: Various atomically dispersed transition metal-nitrogen-carbon catalysts (ADTCs) were synthesized via chemically doping metal ions into ZIF-8 precursor followed by one-step carbonization (Fig. 1a).[1-2] In a typical procedure for synthesize Mn ADTC, 1.314g of 2-mehtylimidazole was dissolved in 15 mL of methanol to form solution A, and 0.167g of $Mn(NO_3)_2{\cdot}4H_2O$ and 0.397g of $Zn(NO_3)_2{\cdot}6H_2O$ were dissolved in 15 mL of methanol to form solution B. Then, solution A was added into solution B dropwise under stirring. The mixture was kept under stirring for 24 h, and the precipitate was separated by centrifugation and washed with methanol for three times. After freeze-drying overnight under vacuum, Mn-ZIF precursor was obtained as powder form. For yielding other metal-ZIF precursors, the same method was used while the molar ratio among Zn and other metal ions was kept constant. Metal doped ZIF-8 precursor was then loaded into a corundum crucible and transferred into a tube furnace with an argon flow for the subsequent pyrolysis. The specific program of pyrolysis is: from 25°C to 900 °C with a ramping rate of 5°C min^{-1}, kept at 900 °C for 2 h under argon atmosphere with a flow rate of 30 mL min^{-1}. After natural cooling, Mn, Fe, Co, Ni and Cu ADTCs were obtained as a black powder. In addition, by utilizing a relatively low-temperature pyrolysis procedure (the pyrolysis temperature was set at 800 °C for 2 h), the Zn ADTC control sample was obtained by the same approach without addition of other transition metal ions. The collected powder was directly used for subsequent physical and electrochemical characterization without any acid treatment.

Physical characterization. High-angle annular dark-field scanning transmission electron microscopy (HAADF-STEM) images and elemental mapping analysis were performed by JEOL JEM-2100F operated at an accelerating voltage of 200 kV. Spherical-aberration-corrected

HAADF-STEM images were obtained by using a Titan 80-300 scanning transmission electron microscope operated at an accelerating voltage of 300 kV, equipped with a probe spherical aberration corrector. X-ray diffraction (XRD) patterns were collected on a Bruker D2 Phaser using monochromatic Cu Kα radiation (λ = 1.54 Å). X-ray photoelectron spectroscopy (XPS) measurements were conducted on a PHI 5000 VersaProbe (ULVAC-PHI, Japan) system using a monochromatic Al Kα X-ray source (1,486.7 eV) with a beam diameter of 100 μm. The C1s peak has been fixed at the binding energy of 284.8 eV. The XPS raw data were fitted by the XPSPEAK41 software using Shirley-type background. Raman spectra were recorded on a LabRAM high resolution Raman spectrometer. Inductively coupled plasma optical emission spectrometry (ICP-OES) analysis was conducted to determine metal content in samples using a NexIon 350 (Perkin Elmer) machine. *Ex situ* metal (Mn, Fe, Co, Ni, Cu and Zn) K-edge X-ray absorption spectra were collected at beamline BL 17C of National Synchrotron Radiation Research Center (NSRRC). Metal (Mn, Fe, Co, Ni, Cu and Zn) L-edge and N K-edge XANES spectra were taken at the BL20A beamline of NSRRC. The corresponding spectra were recorded in total-electron-yield mode.

Operando quick-scanning X-ray absorption spectroscopy. Operando X-ray absorption spectroscopy for metal (Mn, Fe, Co, Ni, Cu and Zn) K-edge were conducted at TPS 44A of NSRRC. A three-electrode system with the same setup for electrochemical measurements was used for the operando measurements. XAS data were recorded in total-fluorescence-yield mode and were collected with an acquisition rate of ~50 s per spectrum to enhance the quality of XANES and EXAFS spectra. The collected data for different metal atoms were calibrated by corresponding metallic foil and were then processed with the ATHENA program. EXAFS analysis was carried out using Fourier transform on k^3-weighted EXAFS oscillations to assess the contribution of each bond pair to Fourier transform peak. The curve fitting of EXAFS spectra was conducted using the software, REX2000, with FEFF8 program.

Electrochemical measurements. All electrochemical measurements were conducted on an electrochemical workstation (Autolab PGSTAT302N) with a standard three-electrode system in 0.5 M $KHCO_3$ solution. Ag/AgCl electrode with 3 M KCl filling solution and a Pt wire were used as the reference and counter electrodes, respectively. For preparing the working electrode, 5 mg of as-synthesized catalyst powder was first dispersed in a mixed solution containing 1mL of ethanol and 20 μL of a 5 wt % Nafion/ethanol solution by sonicating for 30 minutes, then a 50 μL

aliquot of the resulting catalyst ink was loaded on a 0.782 cm^2 glassy carbon electrode. Electrochemical measurements were operated using a homemade H-type cell, where the anode and cathode compartments were separated by an anion exchange membrane (Fumasep FAA-3-PK-130, FUMATECH). Before the measurements, both compartments were filled with 30 mL of electrolytes and were saturated with CO_2 by bubbling the gas for 1 h. For evaluations on CO_2RR properties, chronoamperometry (CA) and linear sweep voltammetry (LSV) measurements were used, where the former measurements were performed at constant potentials in CO_2-saturated 0.5 M $KHCO_3$ solution while the latter were performed in CO_2- and N_2-saturated 0.5 M $KHCO_3$ solution at a scan rate of 10 mV s^{-1}. Potential versus reversible hydrogen electrode (RHE) with *iR* compensation was calculated as $E_{RHE} = E_{Ag/AgCl} + 0.21 + 0.0592 \times pH - iR$. The pH values of CO_2- and N_2-saturated 0.5 M $KHCO_3$ solution used in this work are 7.2 and 8.3, respectively.

The product was collected in the air-tight cell using CA method at cathodic potentials. After the magnitude of charge reaches 5 coulombs, the gaseous products at the cathode compartment was quantified using an Agilent 7890A gas chromatography (GC), which was equipped with a thermal conductivity detector (for H2 and CO) and a flame ionization detector (for hydrocarbons). The calibration curves for CO, H_2, CH_4 and C_2H_4 were obtained using certified standard gas samples (Fig. S20a-d). The liquid products were quantified using a Bruker Advance III 500 MHz nuclear magnetic resonance (NMR) (Fig. S20e). Faradaic efficiency (FE) of each product was calculated according to F.E. (%) = (moles of target products × F × n)/C, where F represents Faraday's constant (96485 C mol^{-1}), n refers to the number of transferred electrons, and C is the total amount of charge passed through the working electrode.

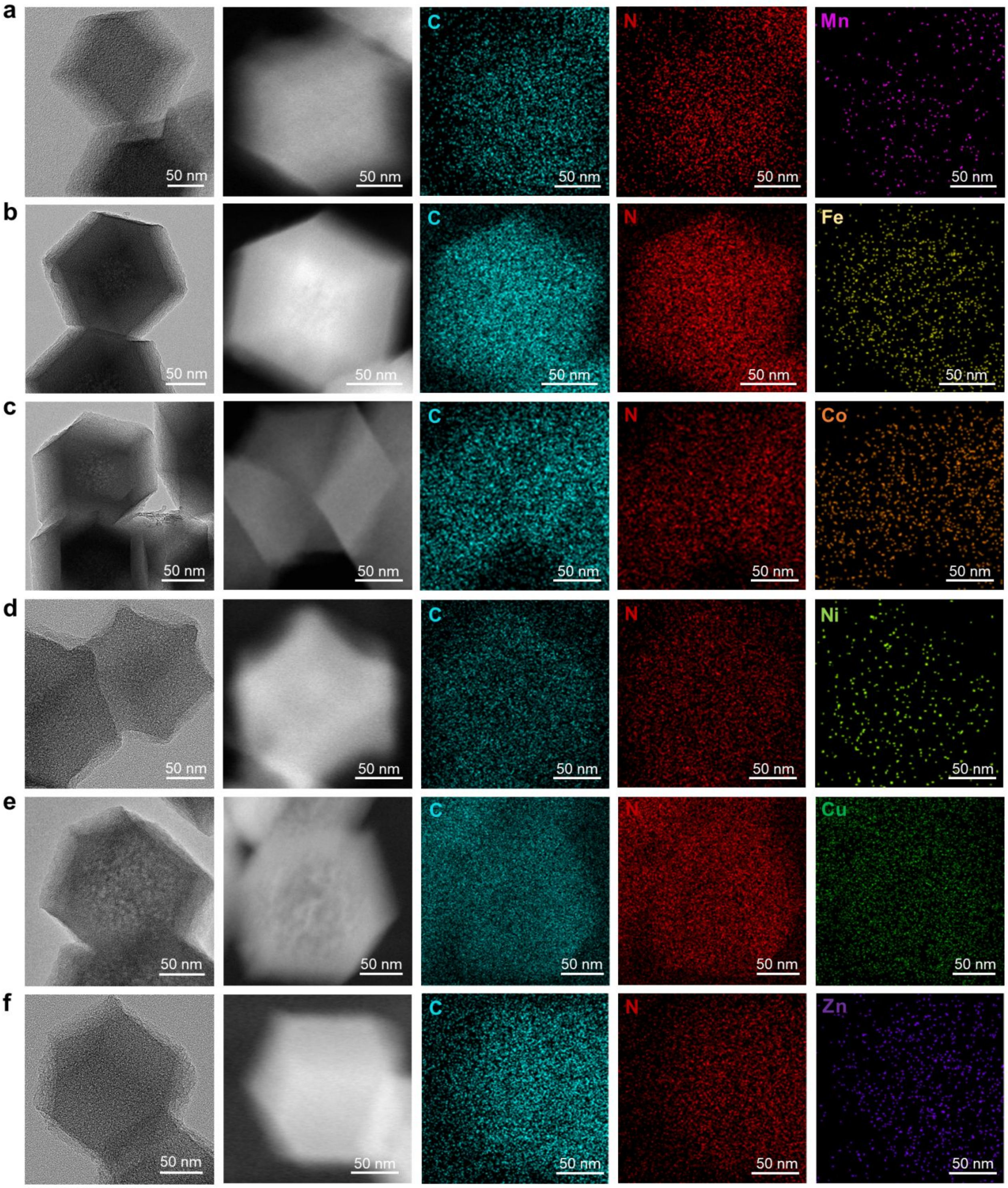

Fig. S1. HRTEM images, HAADF-STEM images and EDS elemental maps for (a) Mn, (b) Fe, (c) Co, (d) Ni, (e) Cu and (f) Zn ADTCs.

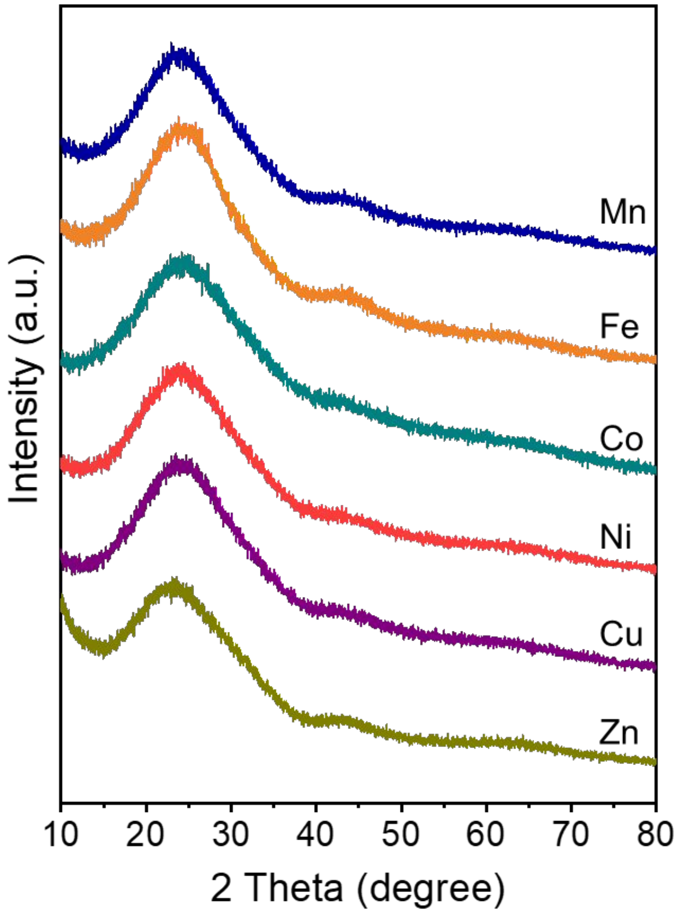

Fig. S2. XRD patterns of Mn, Fe, Co, Ni, Cu and Zn ADTCs. All XRD patterns only show two dominant peaks at 25 and 44˚, corresponding to the characteristic diffractions of graphitic carbon.

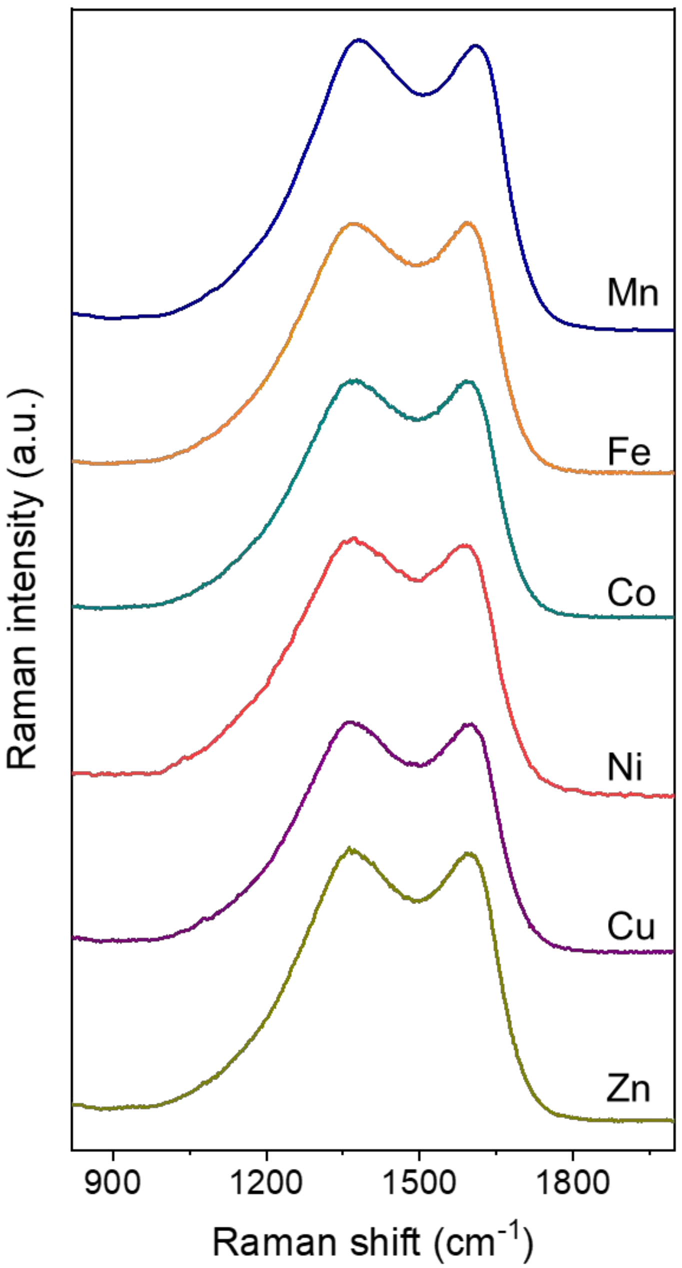

Fig. S3. Raman spectra of Mn, Fe, Co, Ni, Cu and Zn ADTCs. Based on the intensity ratio of D to G band, it reveals that various ADTCs exhibit graphitized carbon structures having the similar defects.

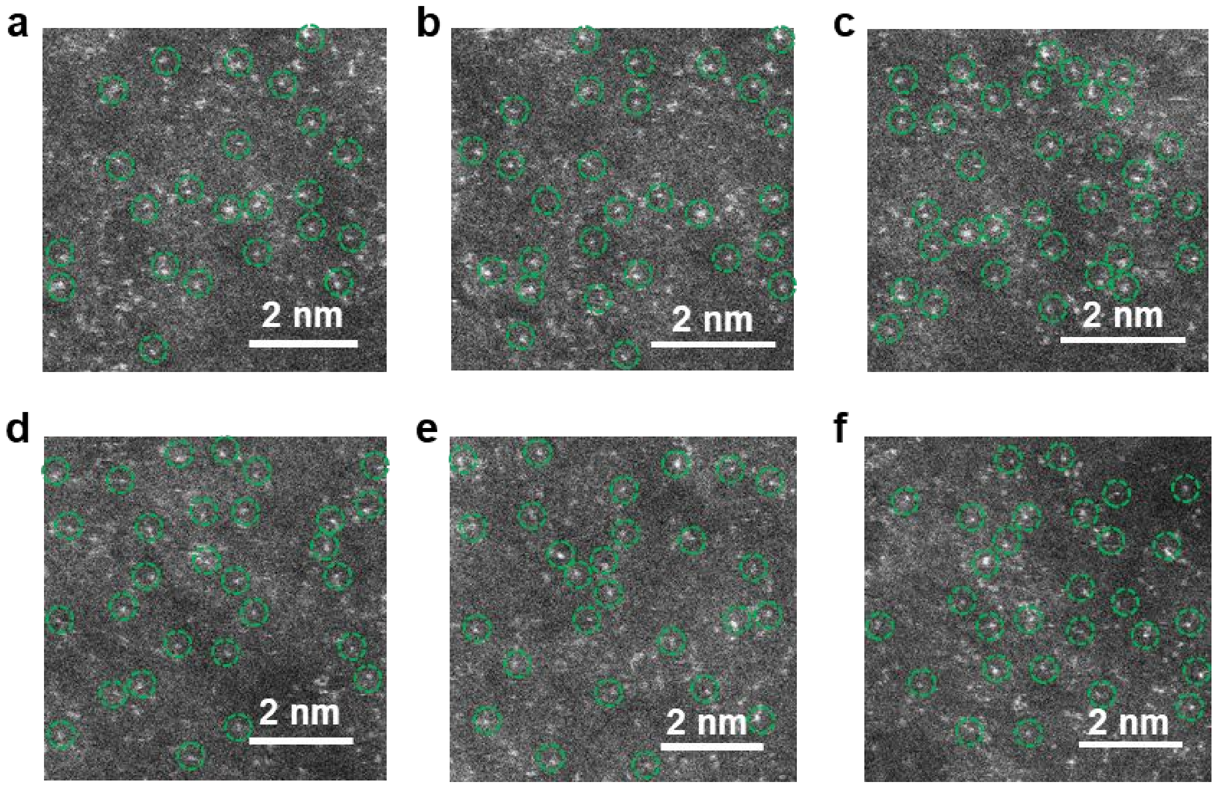

Fig. S4. Aberration-corrected HAADF-STEM images of (a) Mn, (b) Fe, (c) Co, (d) Ni, (e) Cu and (f) Zn ADTCs.

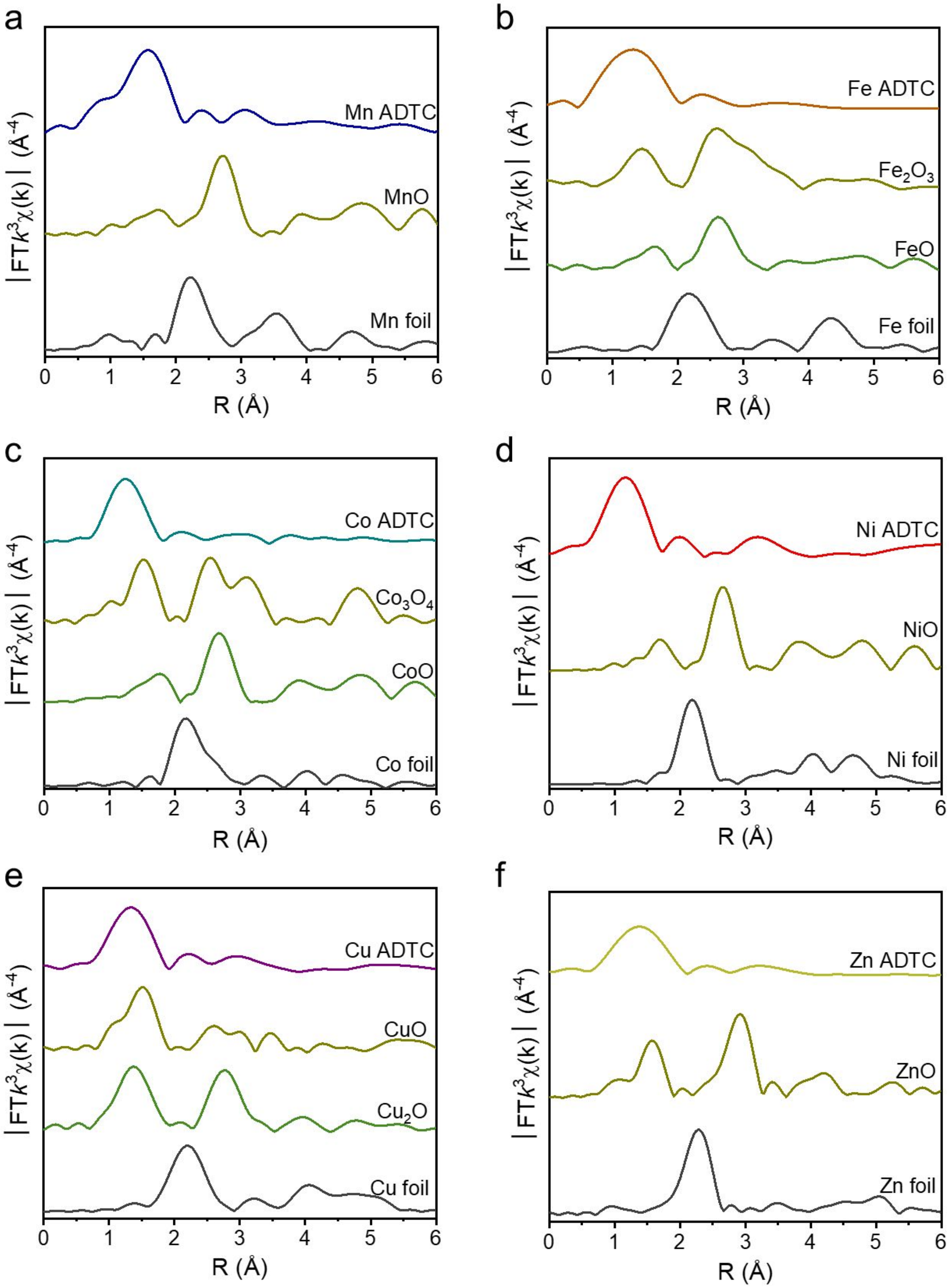

Fig. S5. Metal R-space EXAFS spectra of (a) Mn, (b) Fe, (c) Co, (d) Ni, (e) Cu, and (f) Zn ADTCs, along with corresponding data of reference samples.

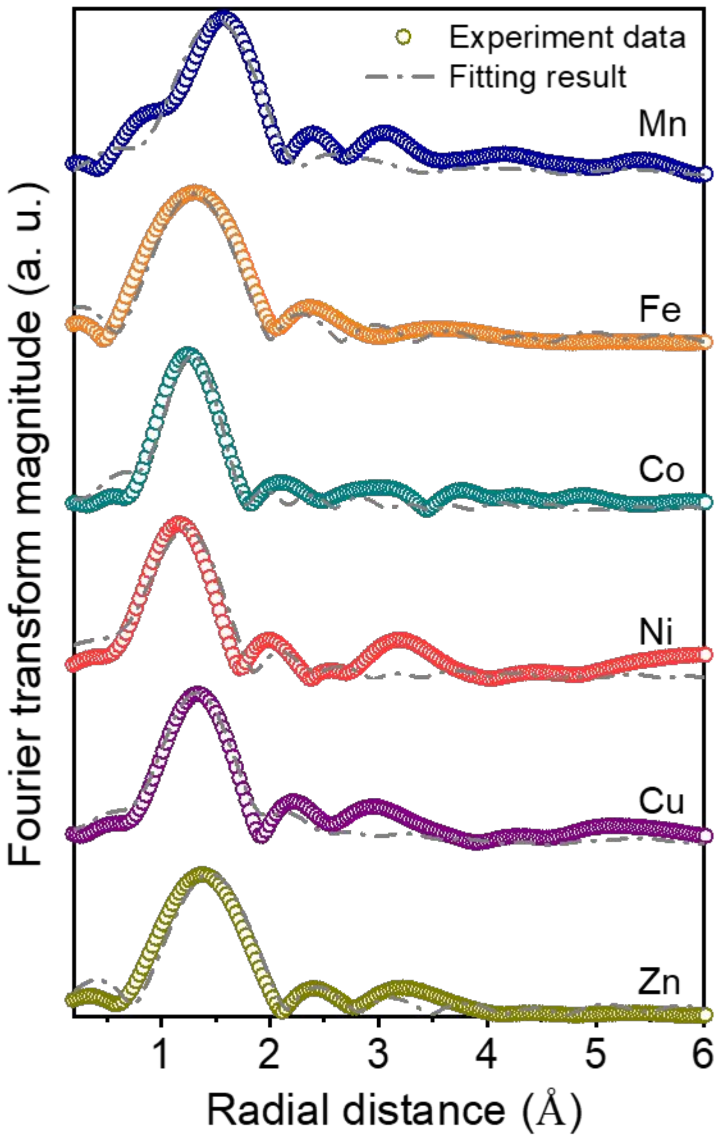

Fig. S6. Metal K-edge EXAFS spectra (hollow circle) and the fitting curves (dash line) for various ADTCs. The fitting results is listed in Table S3.

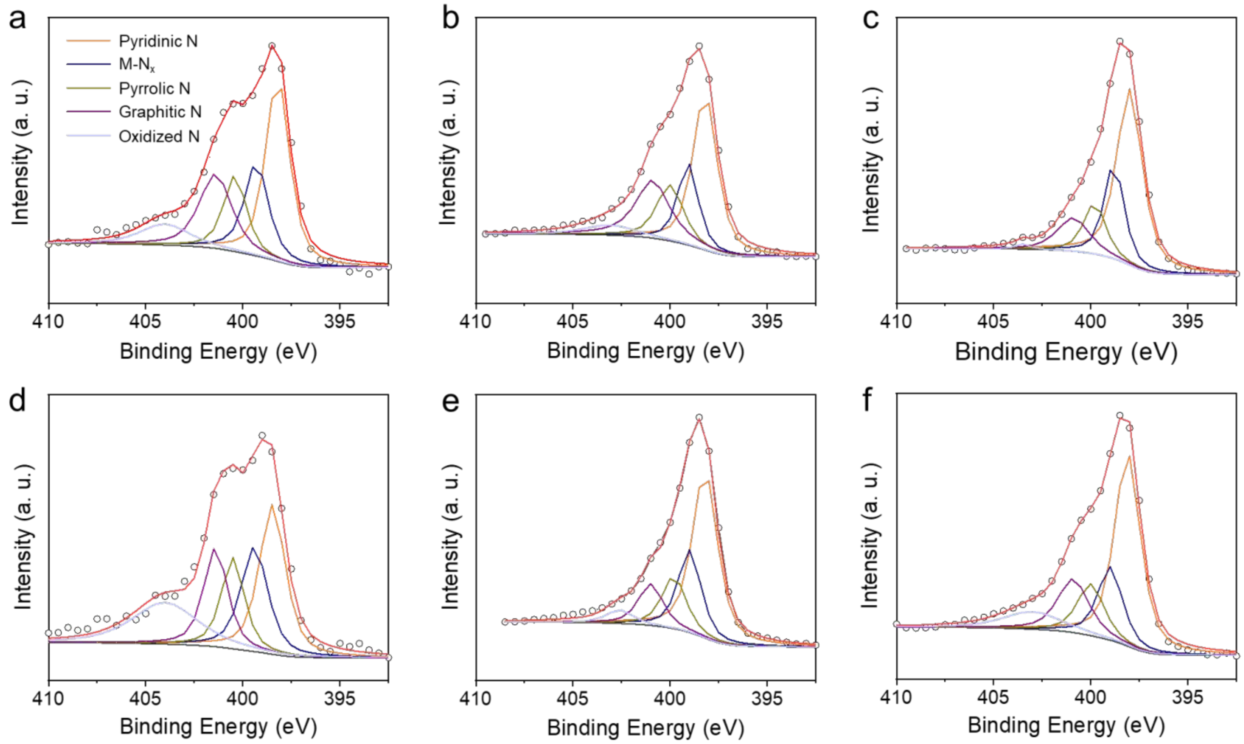

Fig. S7. Deconvoluted N 1s XPS spectra for (a) Mn, (b) Fe, (c) Co, (d) Ni, (e) Cu and (f) Zn. Peaks are assigned to pyridinic N, M-N, pyrrolic N, graphitic N and oxidized N from low energy to high energy.

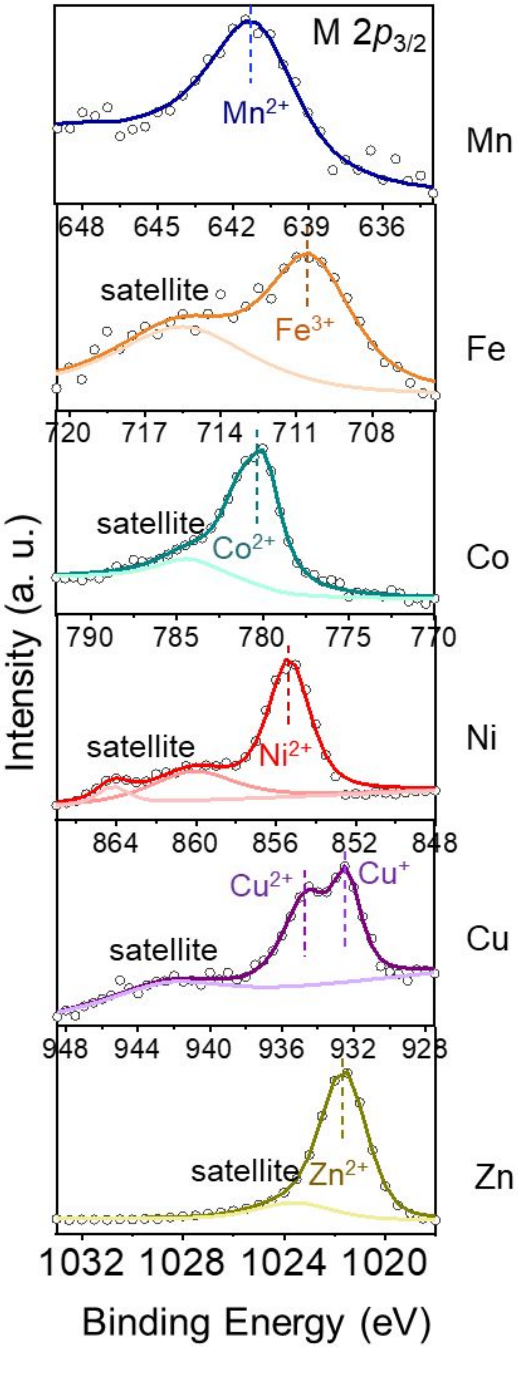

Fig. S8. Deconvoluted metal 2p XPS spectra for various ADTCs.

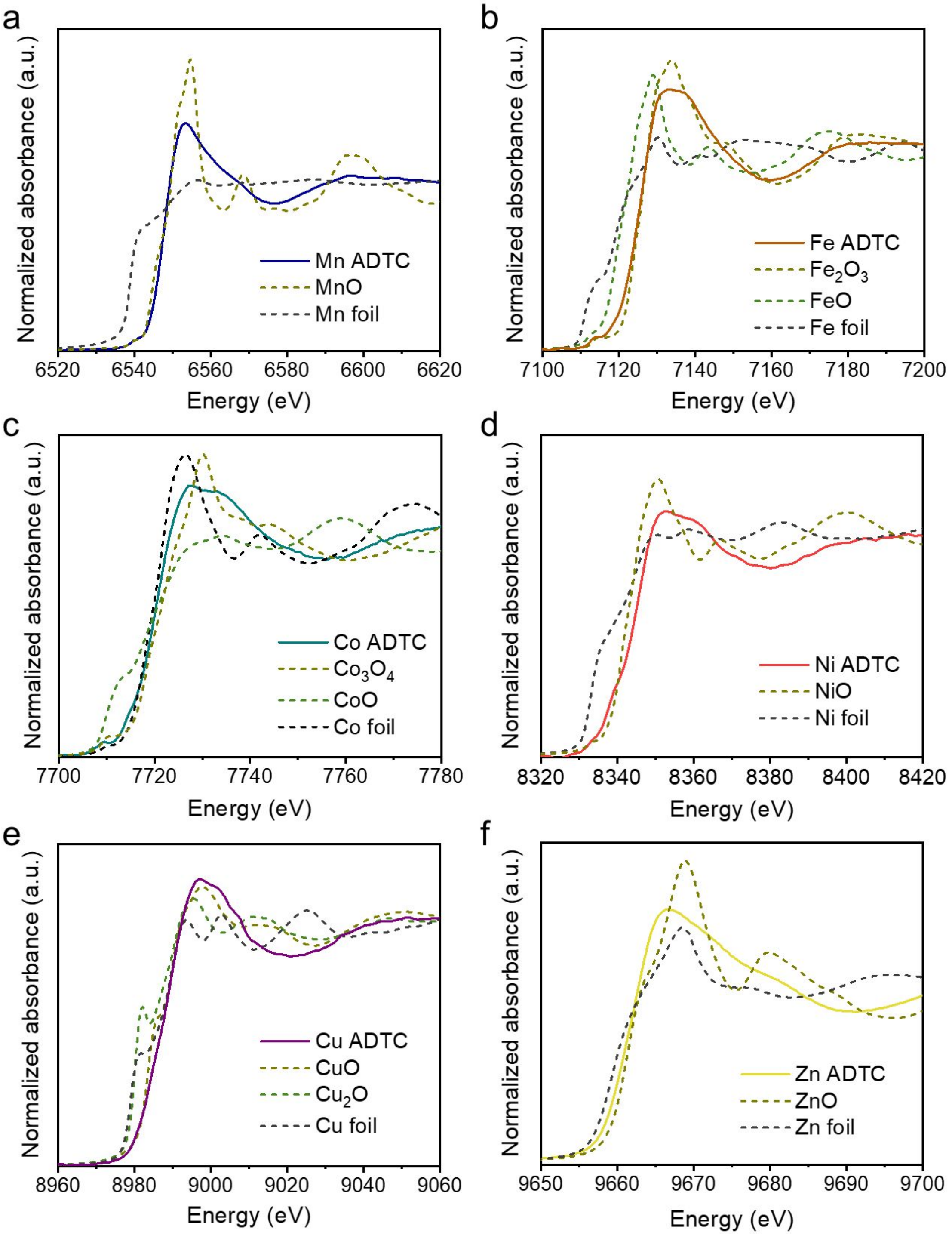

Fig. S9. Metal K-edge XANES spectra of (a) Mn, (b) Fe, (c) Co, (d) Ni, (e) Cu, and (f) Zn ADTCs, along with corresponding data of reference samples.

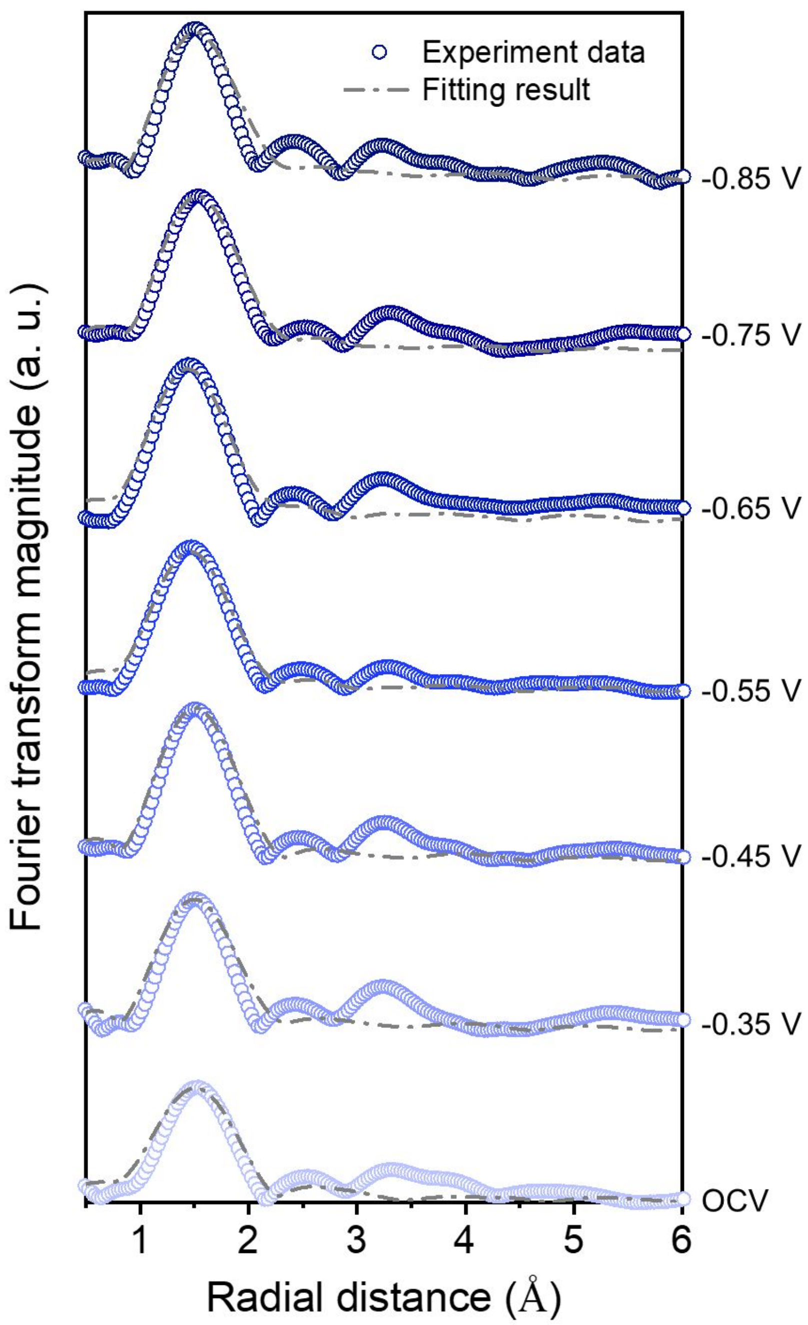

Fig. S10. In situ Fourier-transformed Mn K-edge EXAFS spectra (hollow circle) and the fitting curves (dash line) of Mn ADTC at various applied potentials. The fitting results is listed in Table S4.

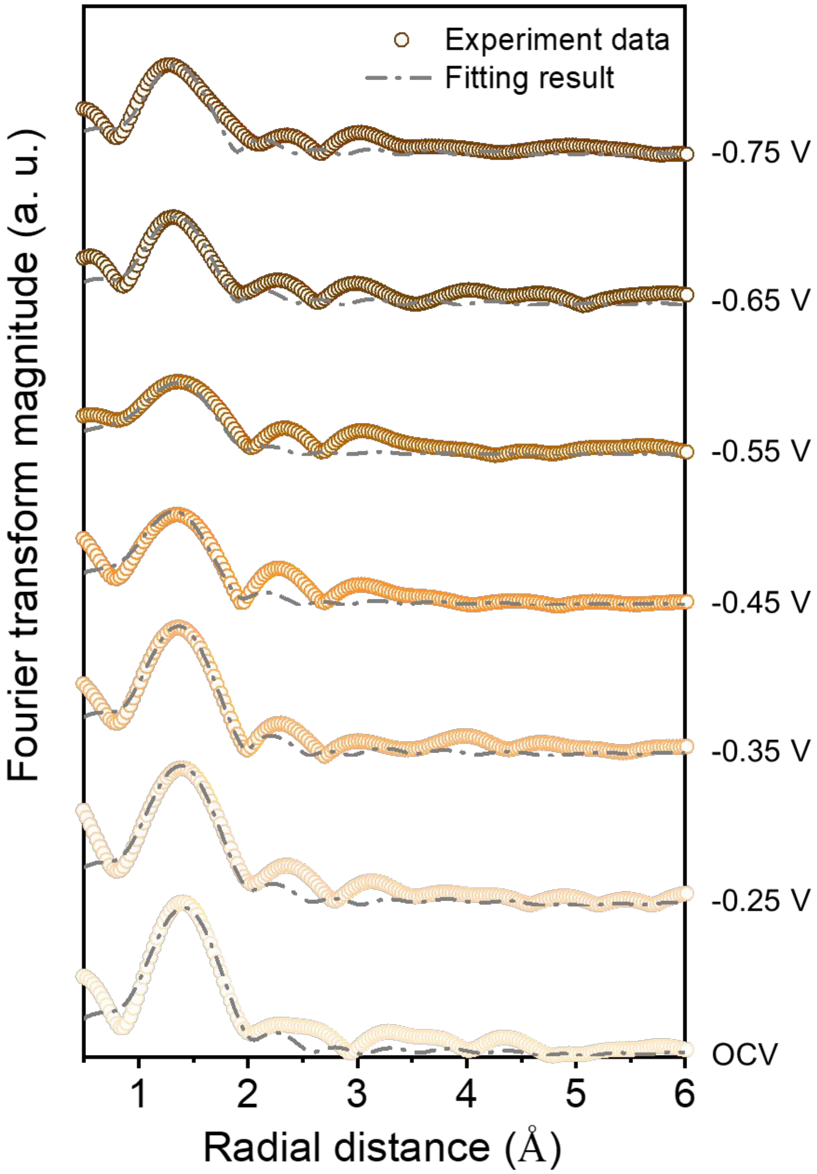

Fig. S11. In situ Fourier-transformed Fe K-edge EXAFS spectra (hollow circle) and the fitting curves (dash line) of Fe ADTC at various applied potentials. The fitting results is listed in Table S5.

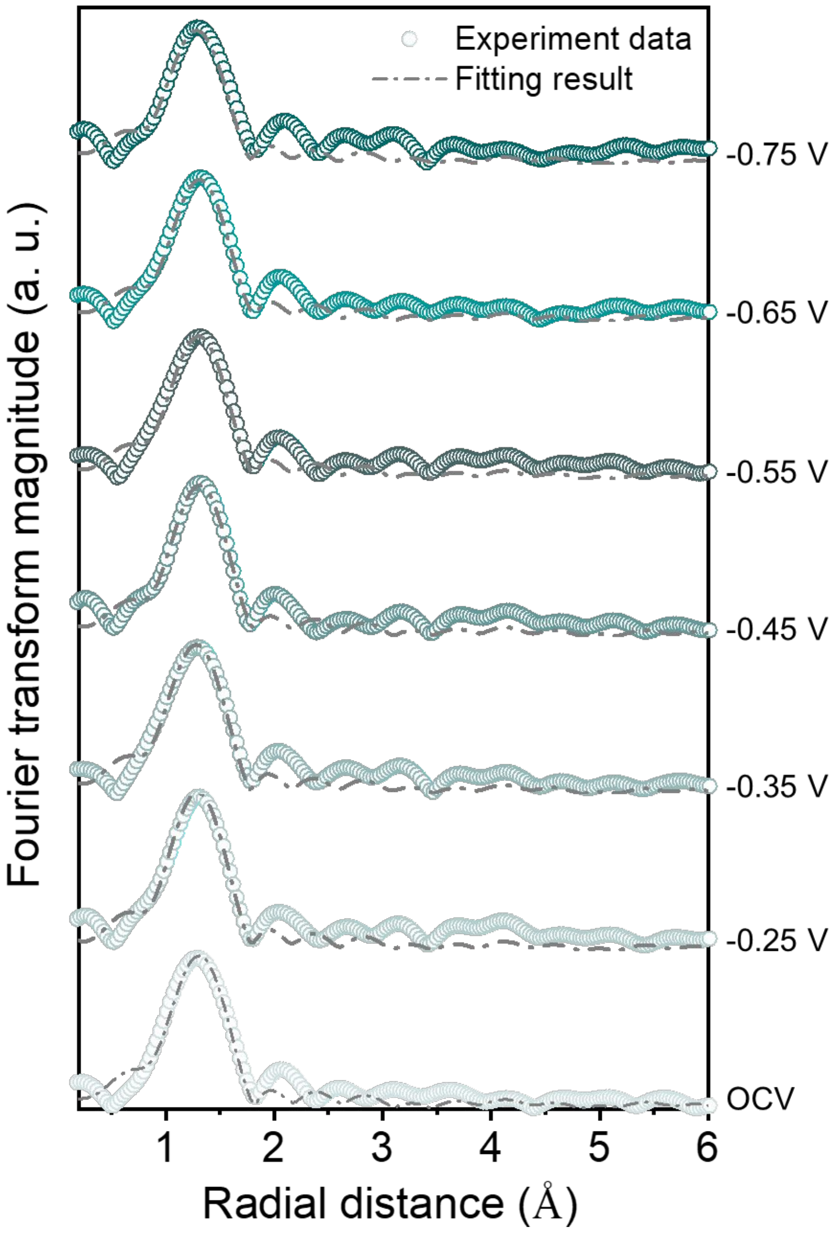

Fig. S12. In situ Fourier-transformed Co K-edge EXAFS spectra (hollow circle) and the fitting curves (dash line) of Co ADTC at various applied potentials. The fitting results is listed in Table S6.

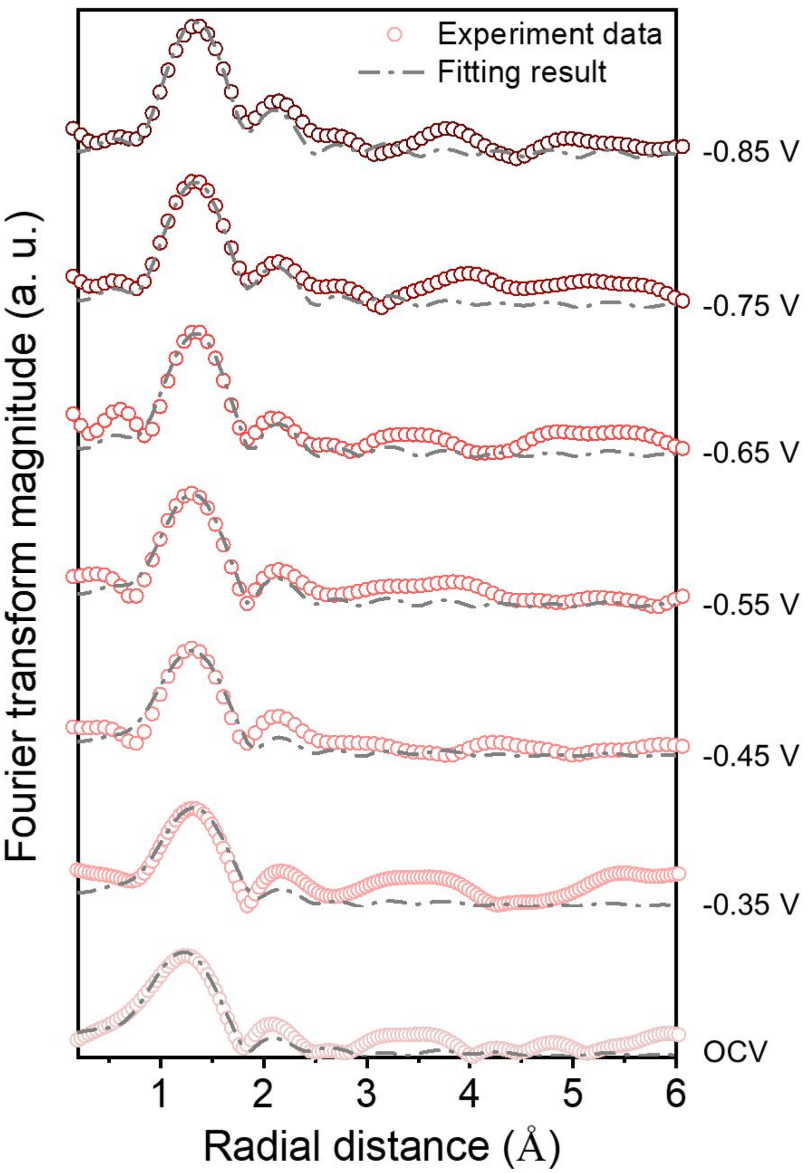

Fig. S13. In situ Fourier-transformed Ni K-edge EXAFS spectra (hollow circle) and the fitting curves (dash line) of Ni ADTC at various applied potentials. The fitting results is listed in Table S7.

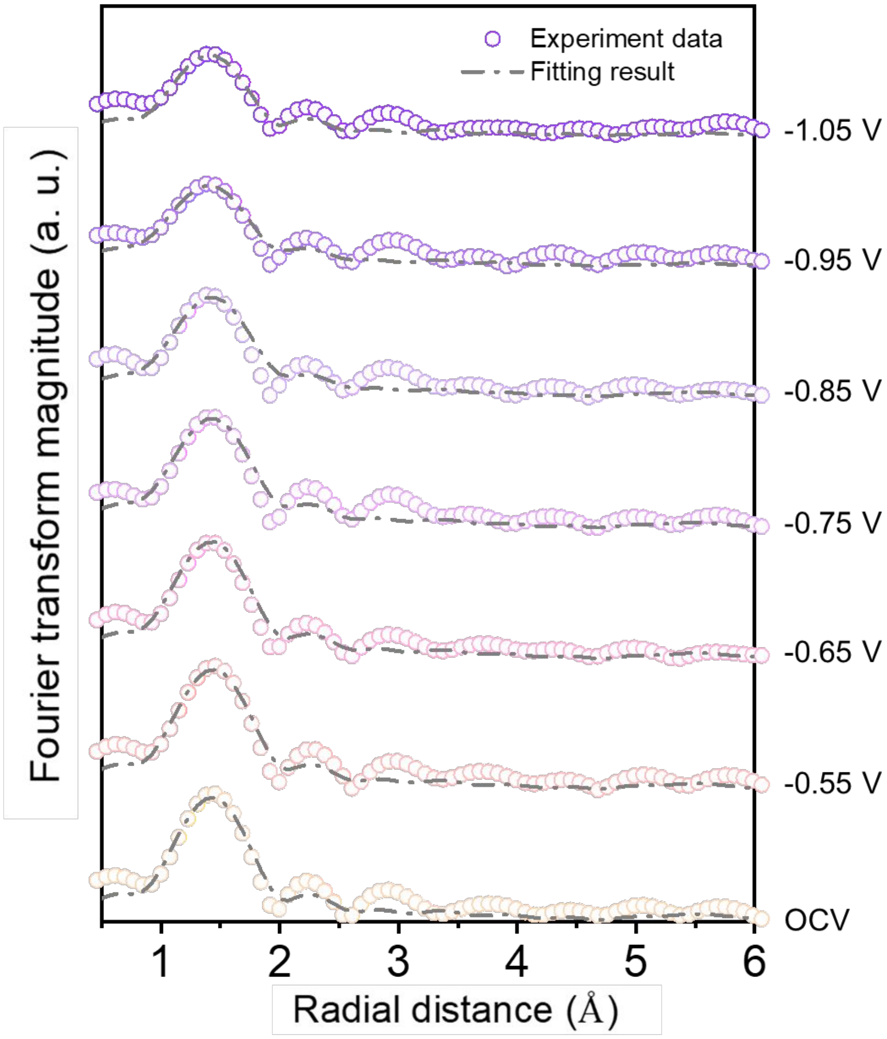

Fig. S14. In situ Fourier-transformed Cu K-edge EXAFS spectra (hollow circle) and the fitting curves (dash line) of Cu ADTC at various applied potentials. The fitting results is listed in Table S8.

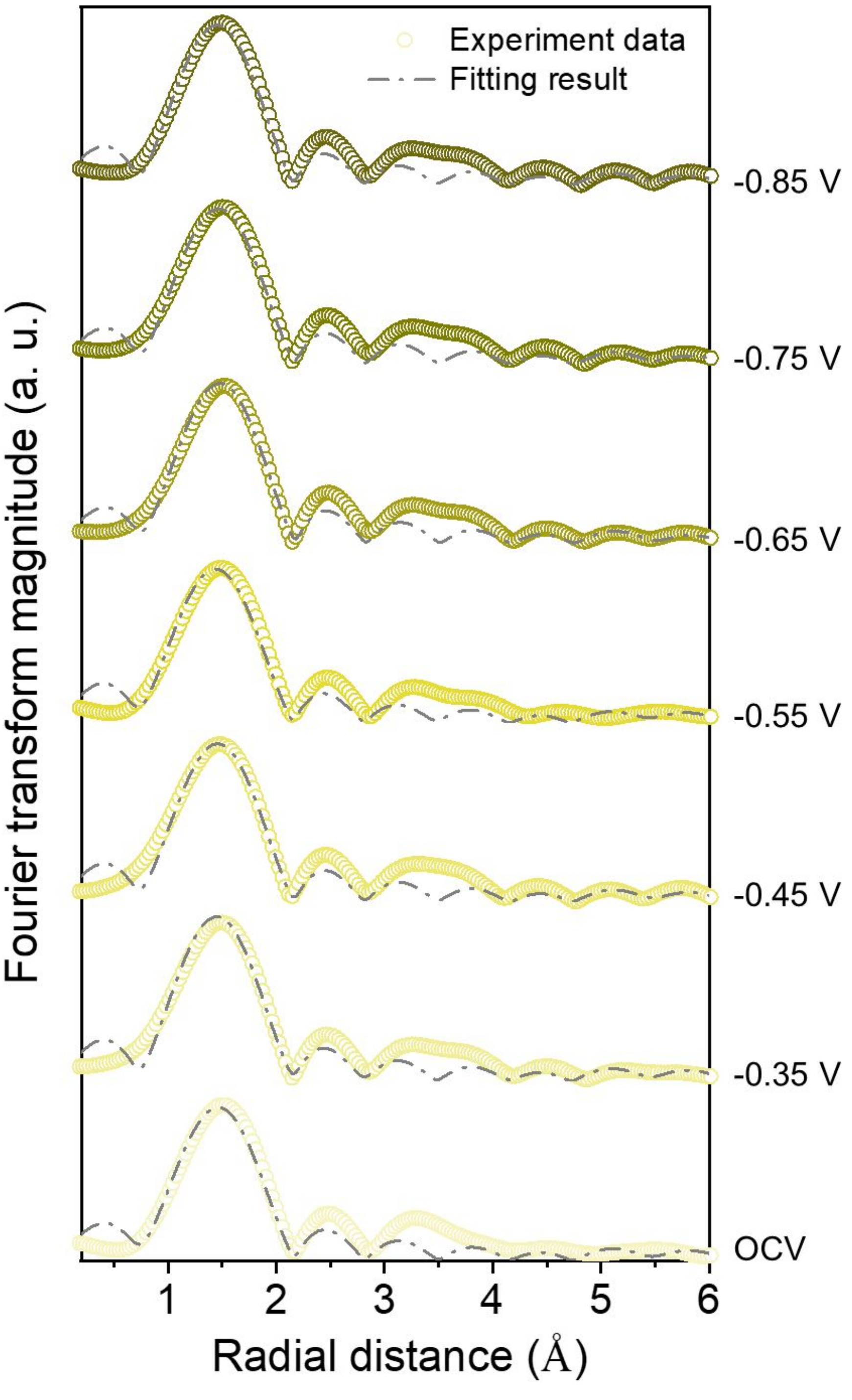

Fig. S15. In situ Fourier-transformed Zn K-edge EXAFS spectra (hollow circle) and the fitting curves (dash line) of Zn ADTC at various applied potentials. The fitting results is listed in Table S9.

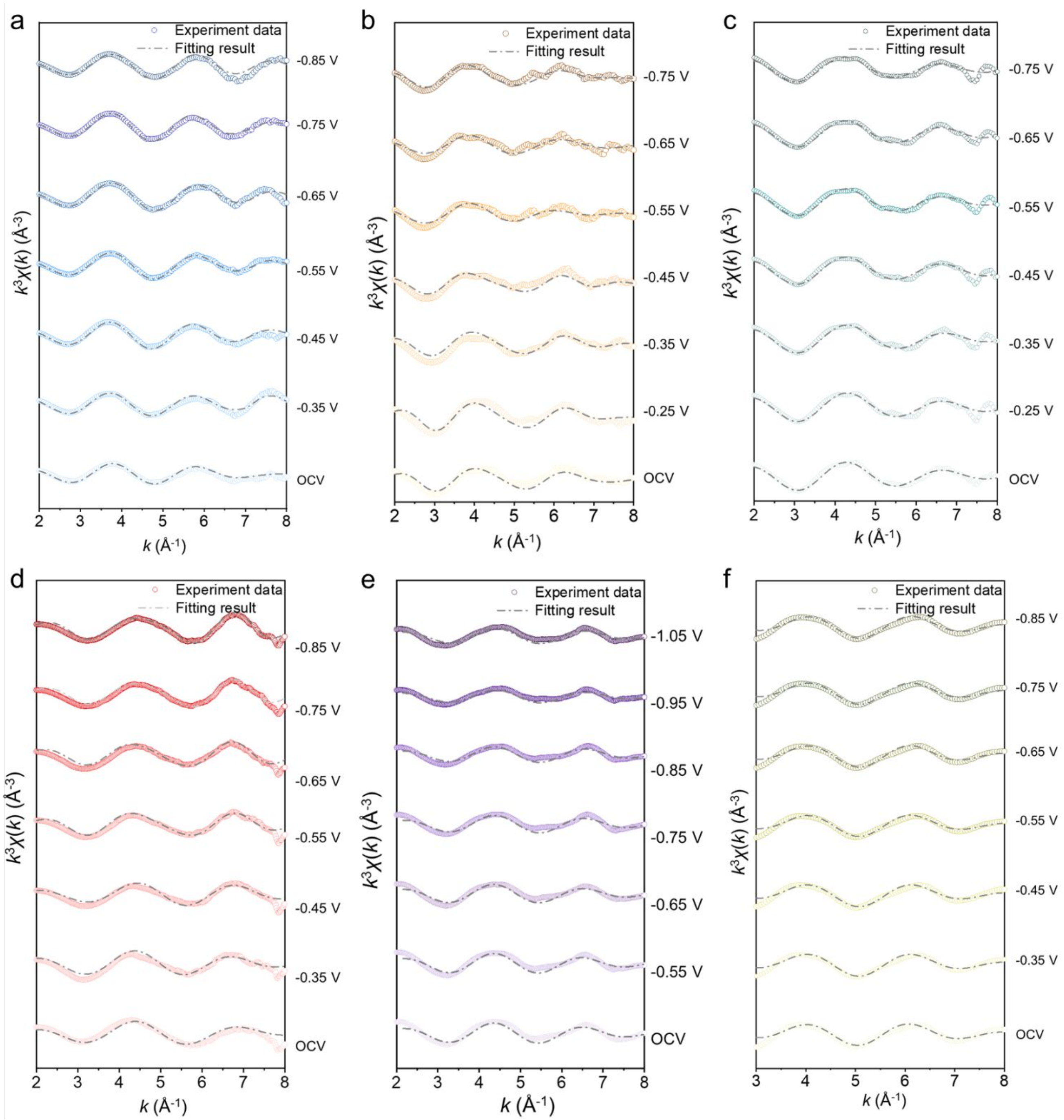

Fig. S16. Fitting results of in situ k-space EXAFS spectra for (a) Mn, (b) Fe, (c) Co, (d) Ni, (e) Cu, and (f) Zn ADTCs.

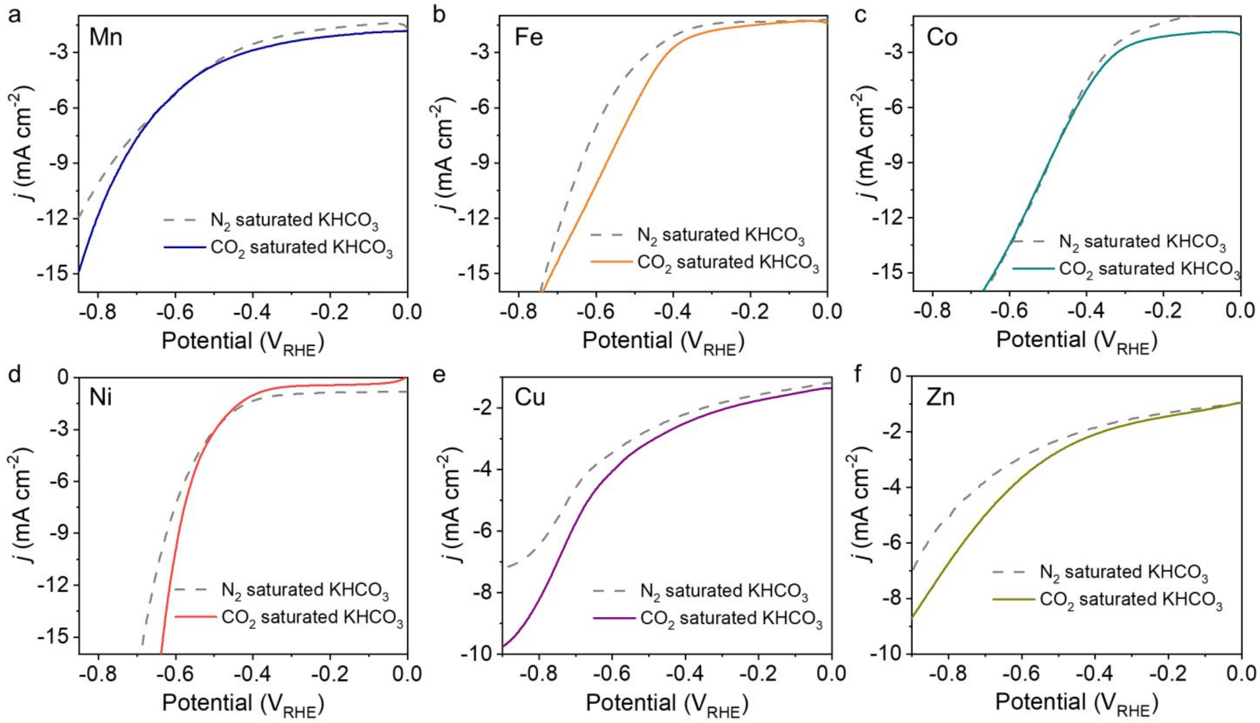

Fig. S17. Linear sweep voltammetry of (a) Mn, (b) Fe, (c) Co, (d) Ni, (e) Cu and (f) Zn ADTCs in CO_2-saturated $KHCO_3$ solution (solid lines) and in N_2-saturated $KHCO_3$ solution (dashed lines).

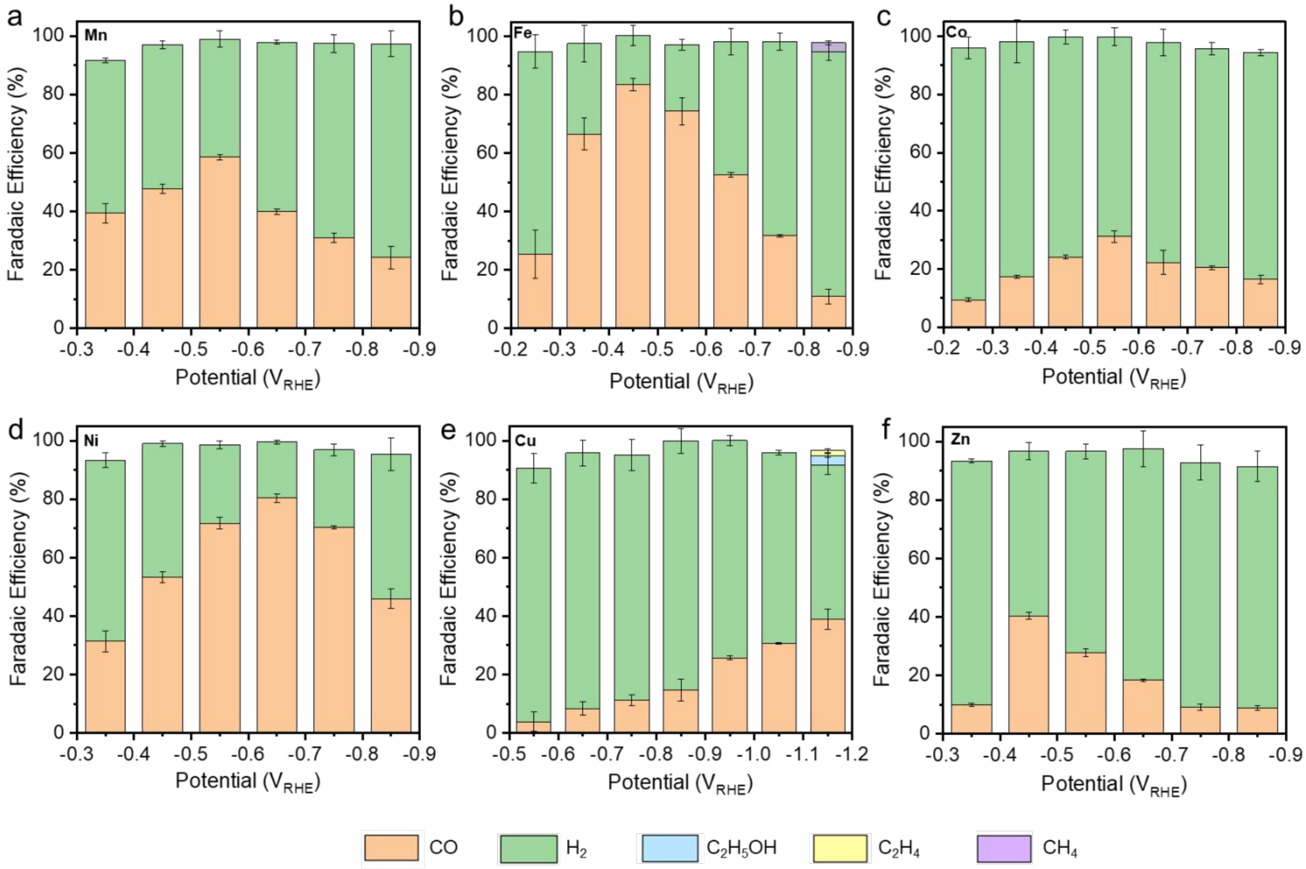

Fig. S18. Potential-dependent selectivity of (a) Mn, (b) Fe, (c) Co, (d) Ni, (e) Cu and (f) Zn ADTCs toward CO_2RR.

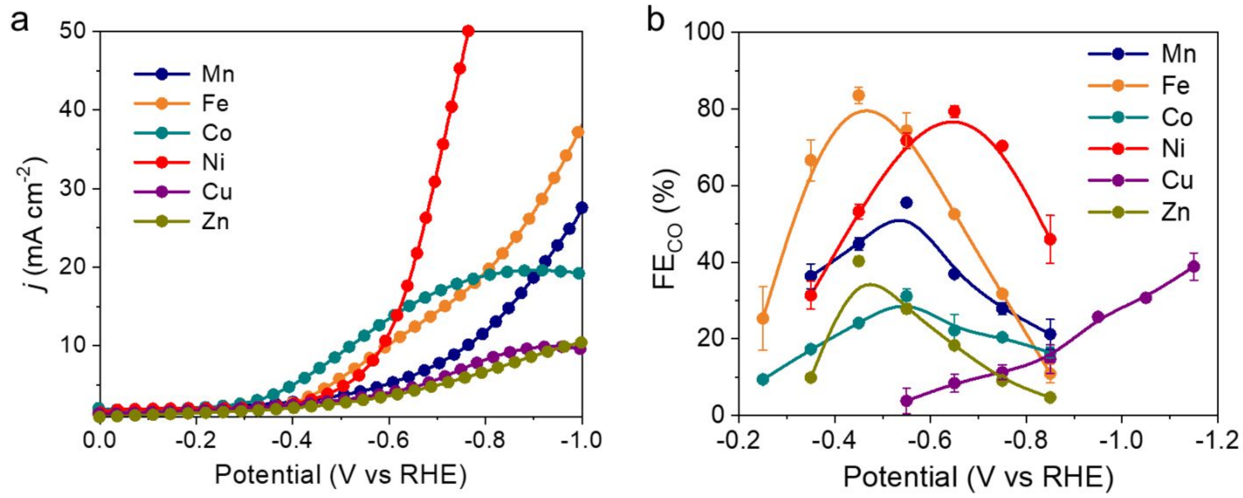

Fig. S19. (a) Linear sweep voltammetry (LSV) curves of various ADTCs recorded in CO_2-saturated 0.5 M $KHCO_3$ electrolyte. (b) Faradaic efficiencies (FE) of CO product for various ADTCs, the error bars represent the standard deviation from three independent measurements.

Note: LSV measurement was performed in CO_2-saturated 0.5 M $KHCO_3$ electrolyte for a preliminary activity evaluation (Fig. S19a). At the low potential range, the current density of ADTCs was observed to follow an increasing trend of Co > Fe > Ni > Mn > Cu > Zn. Note that, such trend was significantly changed with increasing the applied potentials, where Ni ADTC stands out with remarkably enhanced current densities, exhibiting superior activity to that of Fe ADTC. Furthermore, product selectivity over the CO_2RR process was then evaluated under potentiostatic control. Faradaic efficiencies (FE) of CO product on various ADTCs under applied potentials are compared in Fig. S19b. Fe and Ni ADTCs show the highest selectivity of CO_2-to-CO conversion in the studied potential range. At less cathodic potentials, the FE_{CO} of Fe ADTC keeps surpassing that of Ni ADTC and reaches its maximum at the potential of -0.45 V_{RHE}, while starting from -0.55 V_{RHE}, Ni ADTC significantly increases its CO production and exceeds Fe ADTC with the maximum FE_{CO} at -0.65 V_{RHE}. By contrast, Mn, Co, Cu and Zn ADTCs present the limited CO selectivity. Above results demonstrate a promising capability of Ni sites in promoting CO_2-to-CO conversion.

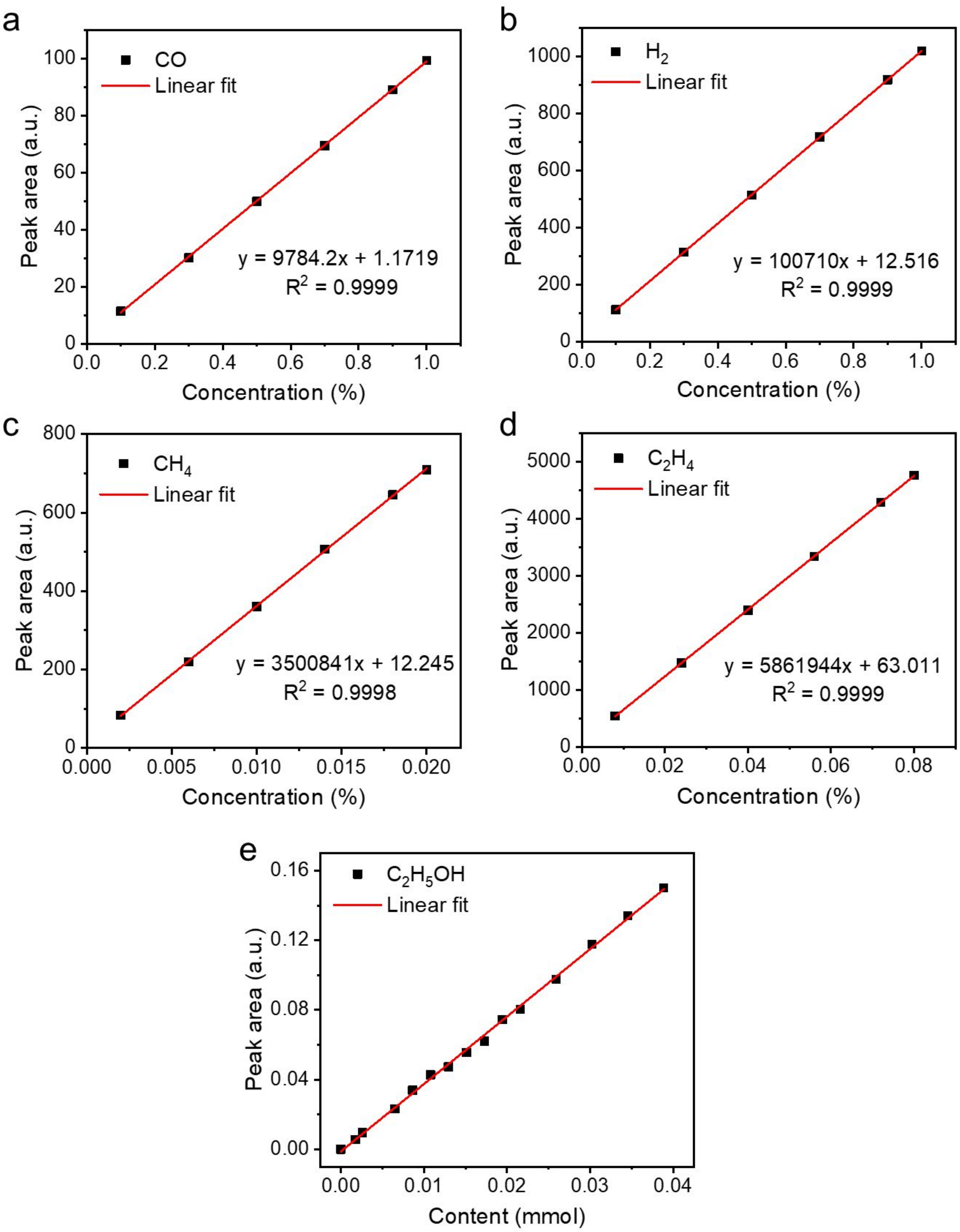

Fig. S20. Calibration curves for the GC analysis of (a) CO, (b) H_2, (c) CH_4, (d) C_2H_4 and NMR analysis of C_2H_5OH.

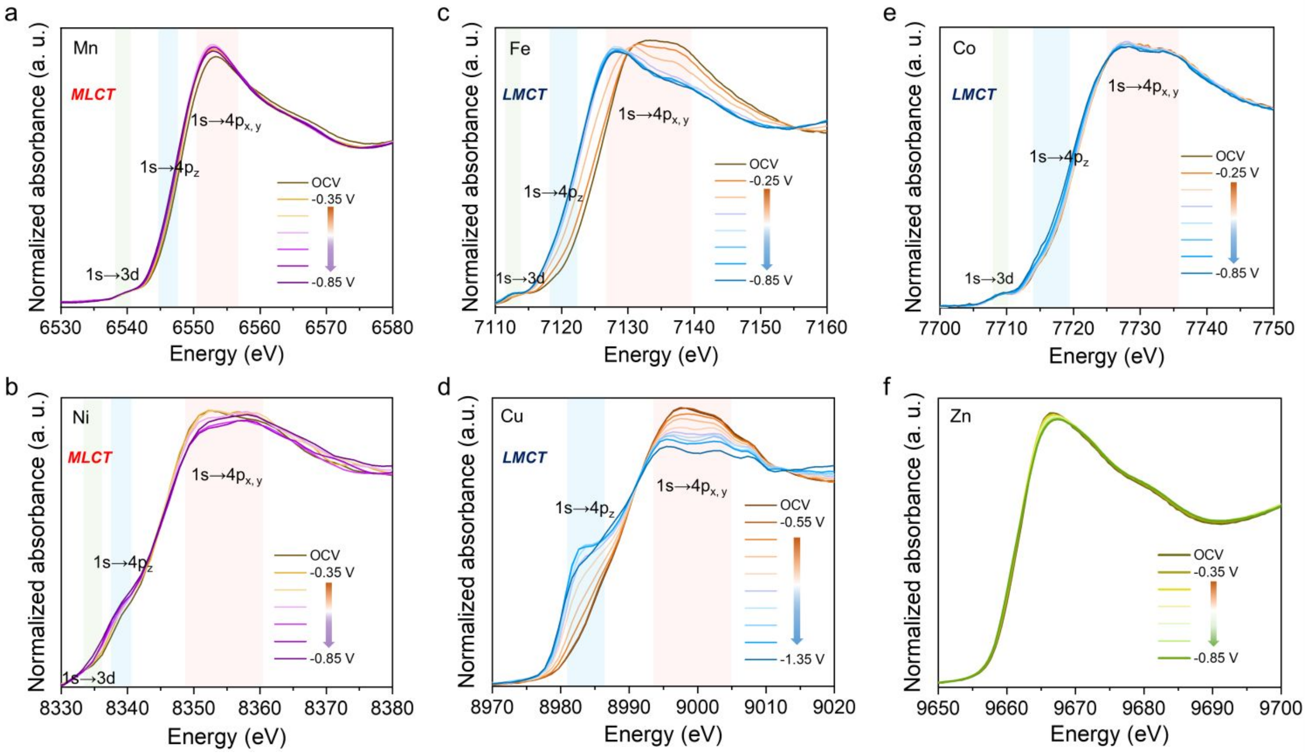

Fig. S21. In situ metal K-edge XANES spectra for (a) Mn, (b) Fe, (c) Co, (d) Ni, (e) Cu and (f) Zn at various applied potentials during CO_2RR.

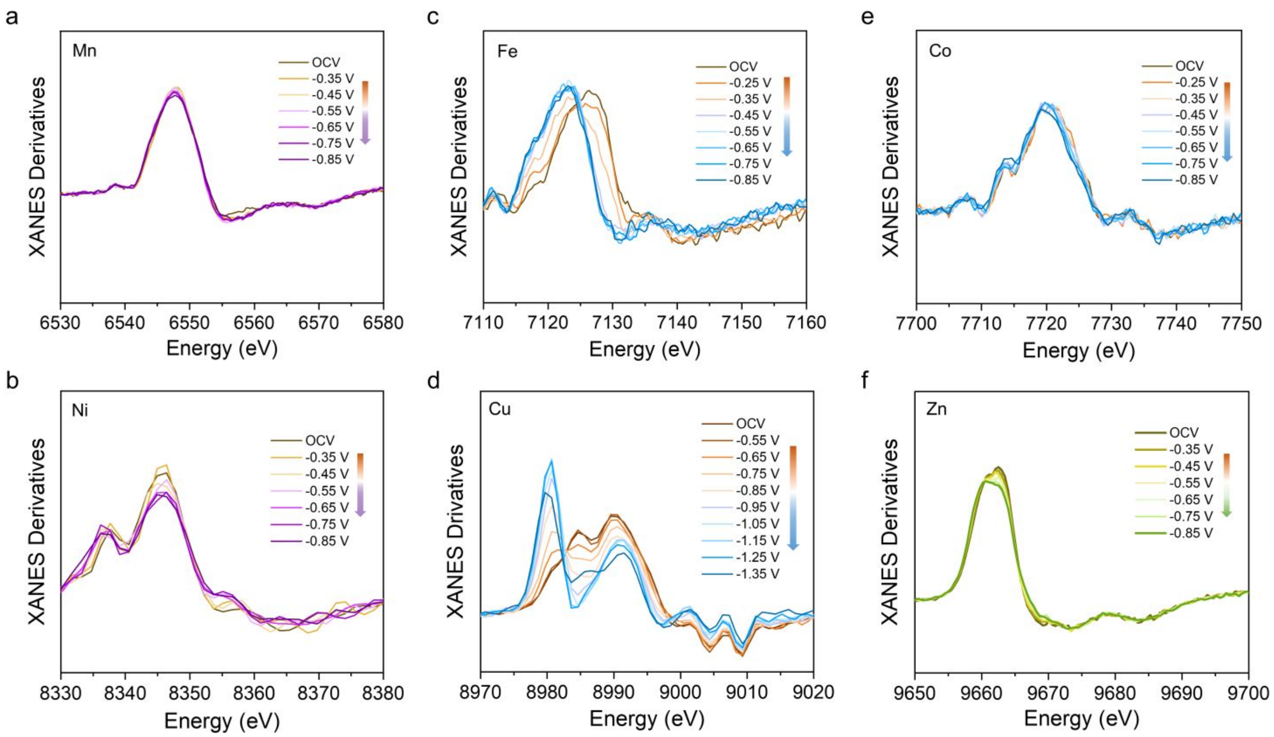

Fig. S22. The first derivative of in situ K-edge XANES spectra for (a) Mn, (b) Fe, (c) Co, (d) Ni, (e) Cu and (f) Zn ADTCs.

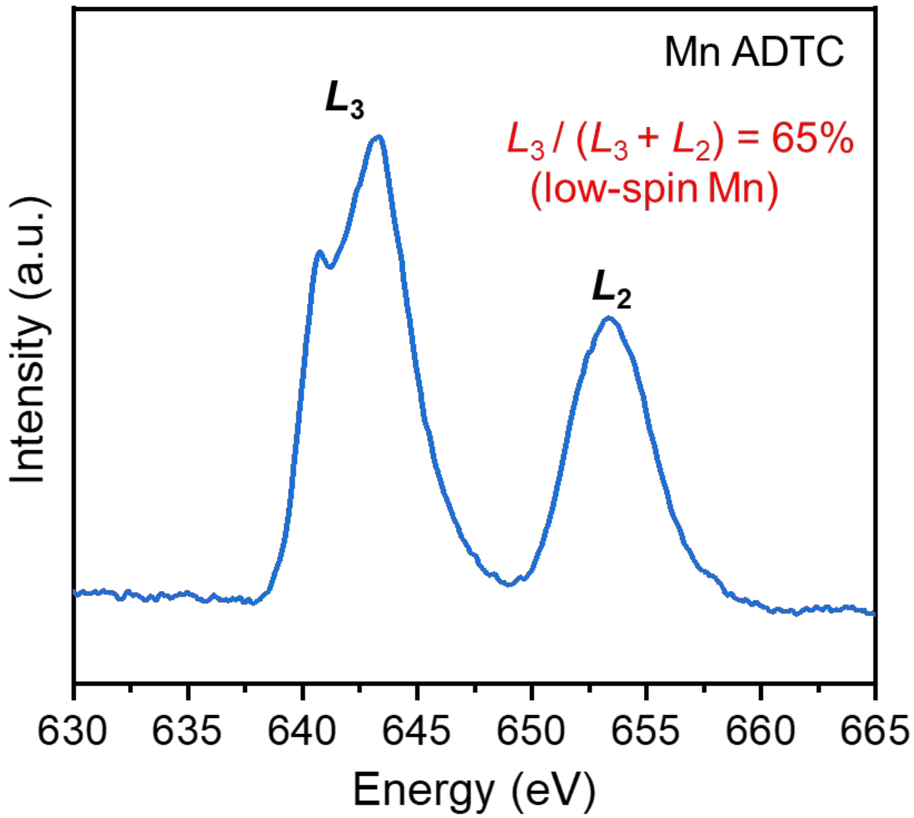

Fig. S23. Mn L-edge XANES spectrum of Mn ADTC after the CO_2RR at -0.85 V_{RHE}.

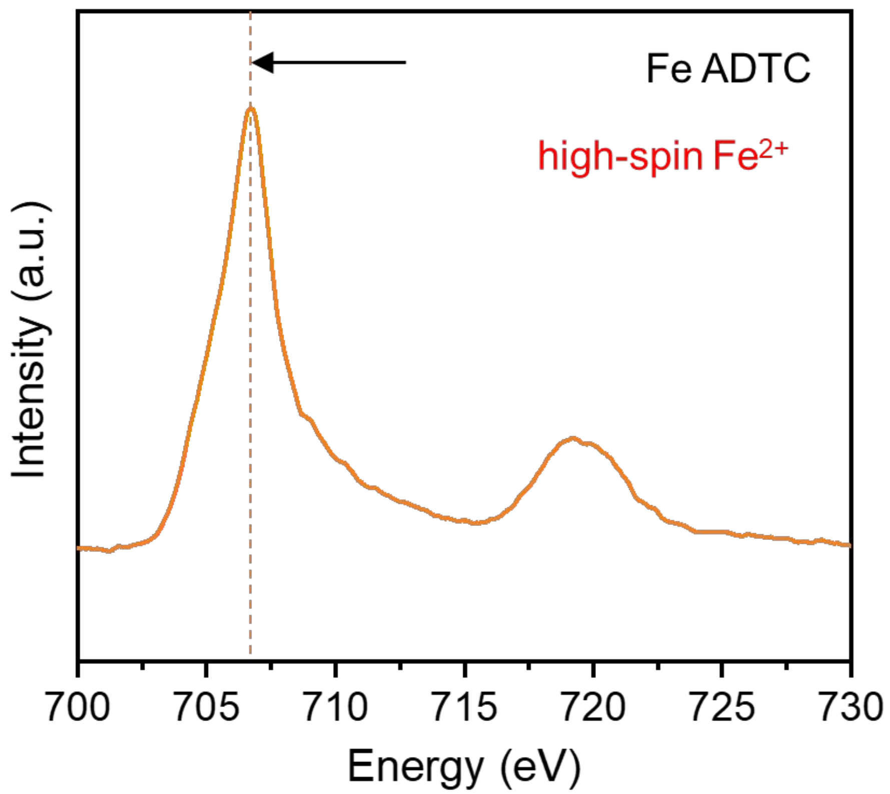

Fig. S24. Fe L-edge XANES spectrum of Fe ADTC after the CO_2RR at -0.65 V_{RHE}.

Table S1. Specific surface area and total pore volume of various ADTCs based on nitrogen physisorption isotherm analysis.

Type of catalysts	BET surface area (m^2 g^{-1})	Total pore volume (cm^3 g^{-1})
**Mn ADTC**	525	0.473
**Fe ADTC**	472	0.441
**Co ADTC**	438	0.461
**Ni ADTC**	386	0.394
**Cu ADTC**	539	0.452
**Zn ADTC**	413	0.359

Table S2. The metal contents in various ADTCs determined by XPS and ICP-OES measurements.

Type of catalysts	XPS analysis (wt %)		ICP-OES analysis (wt %)	
	**M**	**Zn**	**M**	**Zn**
**Mn ADTC**	1.86	0.090	2.03	0.102
**Fe ADTC**	1.96	0.103	2.16	0.096
**Co ADTC**	2.32	0.100	2.49	0.103
**Ni ADTC**	1.99	0.096	2.28	0.104
**Cu ADTC**	1.82	0.089	2.10	0.097
**Zn ADTC**	-	2.36	-	2.55

Table S3. Fitting parameters of M K-edge EXAFS spectra of various ADTCs.

Sample	Path	CN	R (Å)	Δ*E* (eV)	DW ($Å^2$)	R-factor
**Mn ADTC**	Mn-N	3.9 (2)	2.16 (1)	5.6 (8)	0.0091 (2)	1.056
**Fe ADTC**	Fe-N	3.9 (2)	1.96 (1)	-10.2 (6)	0.0078 (4)	1.829
	Fe-O	2.1 (1)	2.07 (2)	3.9 (8)	0.0061 (7)	
**Co ADTC**	Co-N	4.0 (2)	1.88 (2)	-7.6 (8)	0.0085 (4)	1.035
**Ni ADTC**	Ni-N	3.9 (2)	1.85 (2)	-10.5 (7)	0.0107 (5)	2.100
**Cu ADTC**	Cu-N	4.0 (1)	1.95 (1)	2.2 (6)	0.0075 (4)	1.772
**Zn ADTC**	Zn-N	4.1 (1)	1.99 (1)	-2.9 (8)	0.0087 (6)	1.363

CN: coordination number

R: atomic distance

ΔE: energy shift between theoretical and experimental E_0.

DW: Debye-Waller factor

Table S4. Fitting parameters of Mn K-edge EXAFS spectra for Mn ADTC at various potentials during CO_2RR.

	Path	CN	R (Å)	Δ*E* (eV)	DW (Å^2)	R-factor
OCV	Mn-N	4.1 (1)	2.16 (2)	6.2 (7)	0.0107 (2)	2.198
-0.35 V	Mn-N	4.1 (2)	2.13 (2)	3.7 (9)	0.0057 (6)	3.721
	Mn-O	0.6 (2)	2.29 (1)	0.2 (6)	0.0040 (1)	
-0.45 V	Mn-N	4.2 (3)	2.11 (1)	4.2 (6)	0.0045 (3)	4.752
	Mn-O	1.2 (2)	2.30 (2)	-0.3 (3)	0.0046 (4)	
-0.55 V	Mn-N	4.2 (3)	2.08 (2)	2.7 (4)	0.0084 (1)	0.724
	Mn-O	1.7 (2)	2.22 (3)	-3.4 (9)	0.0069 (3)	
-0.65 V	Mn-N	4.2 (3)	2.06 (3)	1.25 (9)	0.0084 (1)	0.930
	Mn-O	1.9 (3)	2.21 (3)	-4.53 (8)	0.0067 (2)	
-0.75 V	Mn-N	4.2 (1)	2.05 (2)	5.9 (5)	0.0087 (1)	1.524
	Mn-O	2.1 (1)	2.21 (3)	-3.0 (8)	0.0135 (2)	
-0.85 V	Mn-N	4.1 (2)	2.05 (1)	7.8 (8)	0.0040 (3)	4.433
	Mn-O	2.1 (2)	2.22 (3)	-3.6 (9)	0.0070 (4)	

CN: coordination number

R: atomic distance

ΔE: energy shift between theoretical and experimental E_0.

DW: Debye-Waller factor

Table S5. Fitting parameters of Fe K-edge EXAFS spectra for Fe ADTC at various potentials during CO_2RR.

	Path	CN	R (Å)	Δ*E* (eV)	DW (Å^2)	R-factor
OCV	Fe-N	3.9 (1)	1.96 (1)	-9.4 (3)	0.0078 (2)	2.148
	Fe-O	2.0 (1)	2.08 (2)	2.1 (5)	0.0076 (6)	
-0.25 V	Fe-N	3.9 (1)	1.96 (1)	-10.1 (8)	0.0078 (1)	2.606
	Fe-O	1.4 (1)	2.10 (3)	2.5 (5)	0.0062 (6)	
-0.35 V	Fe-N	3.9 (1)	1.98 (1)	-10.6 (6)	0.0078 (1)	2.215
	Fe-O	0.9 (2)	2.12 (2)	1.4 (7)	0.0058 (4)	
-0.45 V	Fe-N	3.8 (2)	1.98 (1)	-13.5 (7)	0.0099 (6)	3.400
	Fe-O	0.4 (2)	2.15 (2)	4.6 (9)	0.0079 (8)	
-0.55 V	Fe-N	3.9 (2)	2.00 (1)	-12.6 (9)	0.0108 (7)	2.364
	Fe-O	-	-	-	-	
-0.65 V	Fe-N	3.9 (2)	2.00 (1)	-15.0 (7)	0.0031 (2)	4.163
	Fe-O	-	-	-	-	
-0.75 V	Fe-N	3.5 (2)	2.02 (1)	-15.4 (7)	0.0040 (1)	4.923
	Fe-O	-	-	-	-	

CN: coordination number

R: atomic distance

ΔE: energy shift between theoretical and experimental E_0.

DW: Debye-Waller factor

Table S6. Fitting parameters of Co K-edge EXAFS spectra for Co ADTC at various potentials during CO_2RR.

	Path	CN	R (Å)	Δ*E* (eV)	DW (Å^2)	R-factor
OCV	Co-N	4.1 (1)	1.89 (2)	-5.4 (5)	0.0076 (7)	1.638
-0.25 V	Co-N	4.1 (1)	1.88 (2)	-6.3 (6)	0.0064 (8)	1.961
-0.35 V	Co-N	4.0 (1)	1.87 (2)	-6.8 (7)	0.0071 (8)	2.370
-0.45 V	Co-N	3.9 (1)	1.89 (1)	-5.9 (7)	0.0063 (8)	2.348
-0.55 V	Co-N	3.8 (1)	1.89 (1)	-6.1 (8)	0.0067 (8)	2.899
-0.65 V	Co-N	3.8 (1)	1.89 (1)	-5.9 (9)	0.0060 (8)	2.897
-0.75 V	Co-N	3.4 (1)	1.88 (1)	-7.7 (7)	0.0066 (8)	3.986

CN: coordination number

R: atomic distance

ΔE: energy shift between theoretical and experimental E_0.

DW: Debye-Waller factor

Table S7. Fitting parameters of Ni K-edge EXAFS spectra for Ni ADTC at various potentials during CO_2RR.

	Path	CN	R (Å)	Δ*E* (eV)	DW (Å^2)	R-factor
OCV	Ni-N	3.9 (2)	1.83 (1)	-11.6 (8)	0.0122 (6)	1.221
-0.35 V	Ni-N	3.9 (2)	1.88 (1)	-6.7 (9)	0.0124 (7)	0.690
-0.45 V	Ni-N	3.9 (2)	1.86 (1)	-7.0 (8)	0.0118 (6)	6.100
-0.55 V	Ni-N	4.0 (3)	1.89 (2)	-5.8 (7)	0.0114 (8)	1.712
	Ni-O	0.6 (4)	1.96 (1)	10.1 (4)	0.0030 (9)	
-0.65 V	Ni-N	4.1 (3)	1.92 (1)	-4.0 (4)	0.0105 (1)	2.173
	Ni-O	0.9 (4)	1.97 (1)	-15.5 (1)	0.0053 (2)	
-0.75 V	Ni-N	4.0 (1)	1.92 (1)	-2.8 (5)	0.0098 (1)	0.717
	Ni-O	1.1 (1)	1.96 (2)	11.4 (3)	0.0027 (1)	
-0.85 V	Ni-N	4.0 (2)	1.91 (1)	-3.0 (8)	0.0092 (3)	0.637
	Ni-O	1.2 (2)	1.96 (3)	9.7 (9)	0.0045 (4)	

CN: coordination number

R: atomic distance

ΔE: energy shift between theoretical and experimental E_0.

DW: Debye-Waller factor

Table S8. Fitting parameters of Cu K-edge EXAFS spectra for Cu ADTC at various potentials during CO_2RR.

	**Path**	**CN**	**R (Å)**	**Δ*E* (eV)**	**DW (Å^2)**	**R-factor**
OCV	Cu-N	4.0 (1)	1.94 (2)	-0.1 (3)	0.0080 (3)	0.812
-0.55 V	Cu-N	4.0 (2)	1.94 (3)	0.3 (3)	0.0083 (3)	0.623
-0.65 V	Cu-N	4.1 (2)	1.93 (1)	-0.3 (4)	0.0090 (2)	1.741
-0.75 V	Cu-N	4.0 (3)	1.93 (1)	0.1 (5)	0.0096 (4)	3.856
-0.85 V	Cu-N	3.9 (3)	1.93 (1)	0.1 (7)	0.0102 (3)	3.965
-0.95 V	Cu-N	3.0 (3)	1.95 (1)	-1.0 (5)	0.0097 (3)	1.602
-1.05 V	Cu-N	2.4 (2)	1.96 (4)	-0.9 (6)	0.0070 (6)	0.419

CN: coordination number

R: atomic distance

ΔE: energy shift between theoretical and experimental E_0.

DW: Debye-Waller factor

Table S9. Fitting parameters of Zn K-edge EXAFS spectra for Zn ADTC at various potentials during CO_2RR.

	Path	CN	R (Å)	Δ*E* (eV)	DW (Å^2)	R-factor
OCV	Zn-N	4.2 (2)	1.99 (1)	0.9 (9)	0.0087 (9)	1.229
-0.35 V	Zn-N	4.0 (2)	1.98 (1)	0.6 (7)	0.0079 (9)	2.639
-0.45 V	Zn-N	4.1 (2)	1.98 (1)	1.3 (8)	0.0085 (9)	2.199
-0.55 V	Zn-N	4.2 (3)	1.98 (1)	0.2 (9)	0.0089 (9)	1.162
-0.65 V	Zn-N	4.2 (2)	1.99 (1)	2.4 (6)	0.0084 (9)	1.472
-0.75 V	Zn-N	4.1 (2)	1.99 (1)	2.0 (7)	0.0084 (9)	1.551
-0.85 V	Zn-N	4.1 (2)	1.98 (1)	1.3 (8)	0.0083 (9)	1.092

CN: coordination number

R: atomic distance

ΔE: energy shift between theoretical and experimental E_0.

DW: Debye-Waller factor

References

1. Zhang, H.; Hwang, S.; Wang, M.; Feng, Z.; Karakalos, S.; Luo, L.; Qiao, Z.; Xie, X.; Wang, C.; Su, D.; Shao, Y.; Wu, G. Single Atomic Iron Catalysts for Oxygen Reduction in Acidic Media: Particle Size Control and Thermal Activation. *J. Am. Chem. Soc.* **2017,** *139* (40), 14143-14149.
2. Pan, F.; Zhang, H.; Liu, K.; Cullen, D.; More, K.; Wang, M.; Feng, Z.; Wang, G.; Wu, G.; Li, Y. Unveiling Active Sites of CO2 Reduction on Nitrogen-Coordinated and Atomically Dispersed Iron and Cobalt Catalysts. *ACS Catal.* **2018,** *8* (4), 3116-3122.
